# Supplementary material for: Network embedding unveils the hidden interactions in the mammalian virome
Source: Patterns (N Y). 2023 Apr 24;4(6):100738. doi: 10.1016/j.patter.2023.100738 (PMC10318366; doi:10.1016/j.patter.2023.100738)
Supplement: Document S1. Figures S1–S4 and Tables S1–S3 [file mmc1.pdf]

Patterns, Volume *4*

Supplemental information

Network embedding unveils

the hidden interactions in the mammalian virome

Timothée Poisot, Marie-Andrée Ouellet, Nardus Mollentze, Maxwell J. Farrell, Daniel J. Becker, Liam Brierley, Gregory F. Albery, Rory J. Gibb, Stephanie N. Seifert, and Colin J. Carlson

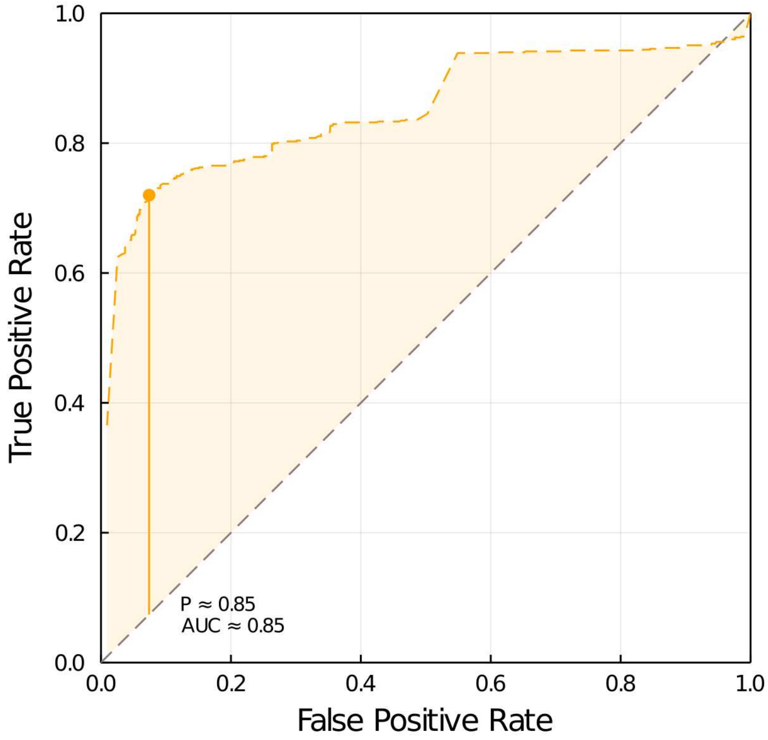

Figure S1: **Receiver operating characteristic (ROC) curve for the best model**. All models (from SVD rank 1 to 20, and using three linear filtering parameterization) have been compared on the same training/validation dataset by measuring the area under the ROC curve. The best model has an area under the ROC curve (AUC) of 0.85. The threshold turning the continuous prediction of LF-SVD into a binary classification was picked to simultaneously maximize the true positive rate and minimize the false positive rate, in practice by maximizing Youden's informedness. By coincidence, this threshold is also approximately equal to 0.85 in the best model.

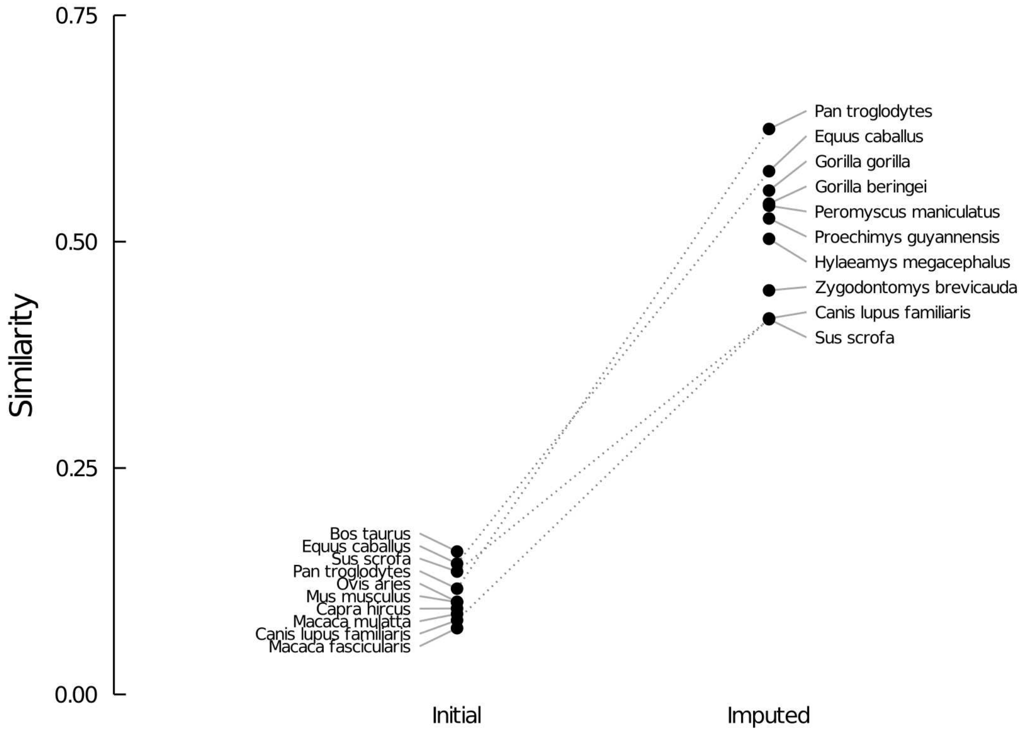

Figure S2: **Pairwise host-similarity to *Homo sapiens* changes post imputation**. The ten hosts with the most viral overlap to *Homo sapiens* (as measured by Jaccard similarity) tend to be livestock. By contrast, the ten most similar hosts after imputation are mostly composed of primates and rodents, which suggests that LF-SVD is able to overcome taxonomic bias in the original dataset.

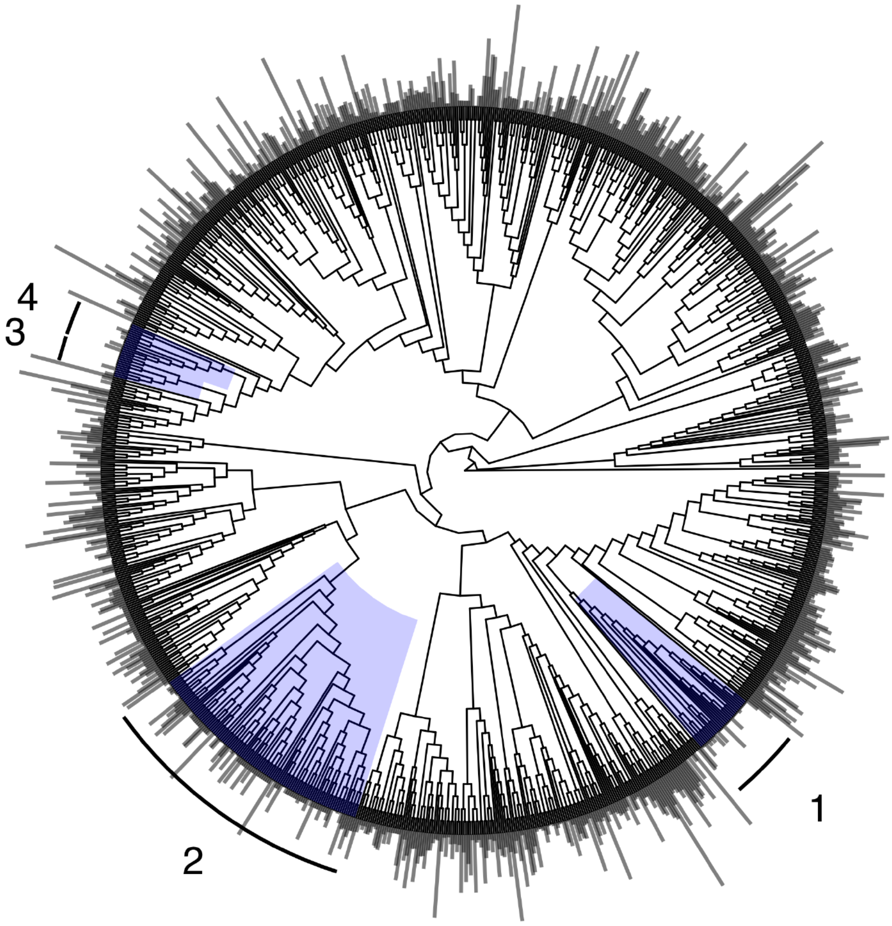

Figure S3: **Phylogenetic bias in missing viruses.** Phylogenetic factorization determined that the majority of species have no phylogenetic signal in the number of missing viruses estimated by the LF-SVD model, with the exception of a handful of small clades that included cetaceans (clade 1), a mostly insectivorous subclade of the Yangochiroptera (clade 2), and two small rodent clades (clades 3 and 4), all of which have significantly fewer than average.

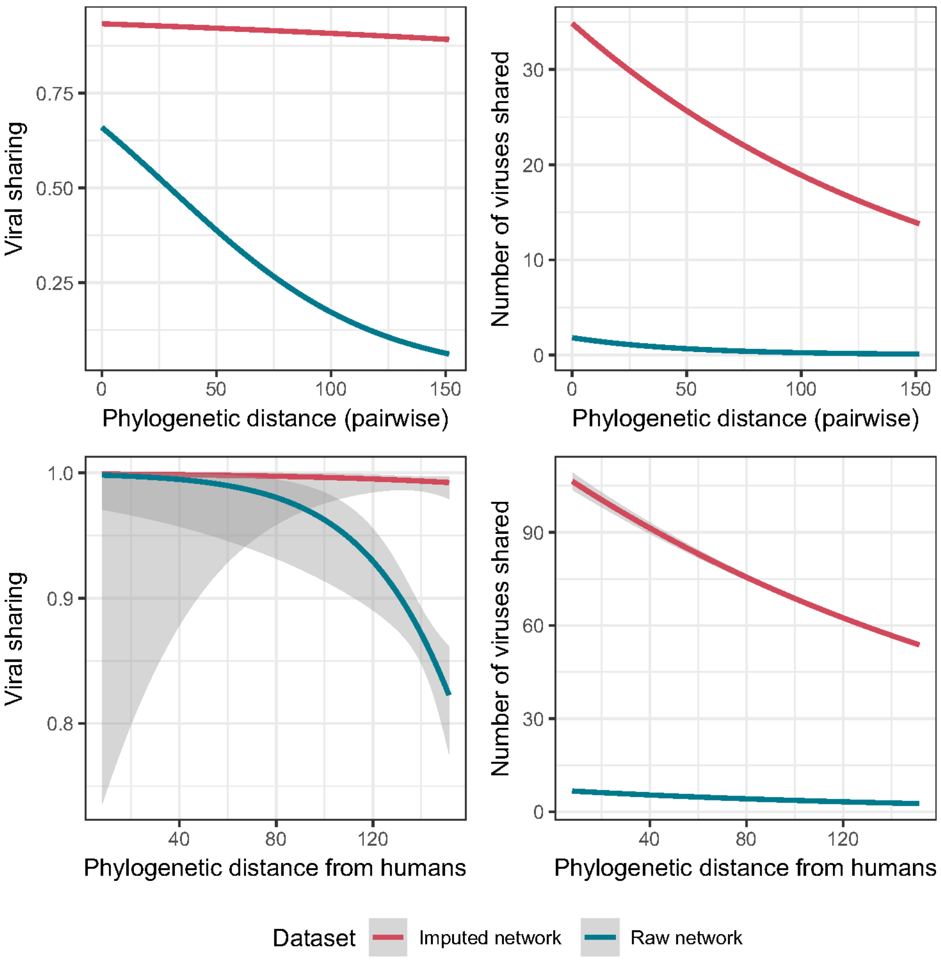

Figure S4: **Evolutionary signals dominate viral sharing.** Phylogenetic distance among all mammals (top) or from humans (bottom) structure viral sharing measured as a binary trait (left) or based on the total number of shared viruses (right). In the imputed network, mosts hosts have many more viruses; as a result, phylogenetic distance is less informative of whether hosts share viruses, because most hosts share at least one virus, but the phylogenetic signal of the count data is much stronger. Curves are given as generalized linear model smooths, with a Poisson distribution for count data and a binomial distribution with a logit link function for viral sharing.

Table S1: **Model performance for the top 10 models by AUC.** Metrics include the AUC and cutoff (expressed as a pseudo-probability), the true positive and true negative rates (TPR, TNR), the positive and negative predictive values (PPV, NPV), the false negative and positive rates (FNR, FPR), the false discovery and false omission rates (FDR, FOR), the critical success index (CSI), accuracy (ACC), and Youden's J.

model	rank	AUC	cutoff	TPR	TNR	PPV	NPV
connectance	12	0.849	0.846	0.72	0.925	0.906	0.769
connectance	11	0.846	0.908	0.684	0.936	0.914	0.75
connectance	17	0.844	0.929	0.692	0.935	0.913	0.754
connectance	8	0.842	0.705	0.701	0.895	0.868	0.751
hybrid	12	0.841	0.707	0.703	0.877	0.851	0.748
connectance	14	0.839	0.902	0.7	0.929	0.907	0.758
hybrid	11	0.837	0.82	0.647	0.918	0.888	0.723
connectance	5	0.836	0.931	0.66	0.94	0.916	0.735
connectance	7	0.836	0.948	0.655	0.957	0.939	0.735
connectance	16	0.835	0.961	0.667	0.945	0.923	0.741

(Continued:)

model	rank	FNR	FPR	FDR	FOR	CSI	ACC	J
connectance	12	0.28	0.074	0.093	0.23	0.669	0.823	0.645
connectance	11	0.315	0.063	0.085	0.25	0.643	0.811	0.621
connectance	17	0.307	0.064	0.086	0.245	0.649	0.814	0.627
connectance	8	0.298	0.104	0.131	0.248	0.634	0.798	0.596
hybrid	12	0.296	0.122	0.148	0.251	0.626	0.79	0.581
connectance	14	0.299	0.07	0.092	0.241	0.653	0.815	0.629
hybrid	11	0.352	0.081	0.111	0.276	0.598	0.783	0.566
connectance	5	0.339	0.059	0.083	0.264	0.623	0.8	0.6
connectance	7	0.344	0.042	0.06	0.264	0.628	0.806	0.613
connectance	16	0.332	0.054	0.076	0.258	0.632	0.807	0.613

Table S2: **Imputation reduces the effect of sampling bias.** To explore whether network imputation via LF-SVD is extrapolating existing research biases, we conducted a set of comparative analyses investigating the how the explanatory power of sampling effort on viral species richness changes after network imputation. We find that after imputation, the slope of the relationship (β) decreases, and sampling effort explains less of the variance in viral richness (R^2), suggesting that imputation via LF-SVD is not merely recapitulating the observed sampling effort per host. Statistics are given for a phylognetic generalized linear model fit with the maximum likelihood estimate of Pagel's λ. Predictors and responses were log-10 transformed prior to analyses.

Viral richness	Publications	β	S.E.	R^2	λ	λ 95% CI
Observed	Cites (all)	0.53	0.02	0.46	0.59	(0.47-0.69)
LF-SVD imputed	Cites (all)	0.39	0.02	0.23	0.59	(0.45-0.72)
Observed	Cites (virus-related)	0.71	0.02	0.54	0.45	(0.31-0.58)
LF-SVD imputed	Cites (virus-related)	0.47	0.03	0.22	0.60	(0.46-0.71)

Table S3: **Phylofactorization of missing viruses.** Significant clades identified from a phylogenetic factorization of missing virus counts. Included taxa are listed alongside the number of species and the mean number of missing viruses for each clade in comparison to the paraphyletic remainder. Clade codes match ED Figure S3.

c

Clade	Included taxa	n	clade
1	Ziphiidae, Physeteridae, Phocoenidae, Monodontidae, Delphinidae, Eschrichtiidae, Balaenopteridae, Balaenidae	30	18
2	Nycteridae, Emballonuridae, Natalidae, Molossidae, Vespertilionidae	109	43
3	*Calomys*, *Graomys*, *Phyllotis*, *Loxodontomys*, *Abrothrix*	11	11
4	*Bibimys*, *Oxymycterus*, *Necromys*, *Akodon*, *Thaptomys*	15	16
