# Supplementary material for: Network embedding unveils the hidden interactions in the mammalian virome
Source: Patterns (N Y). 2023 Apr 24;4(6):100738. doi: 10.1016/j.patter.2023.100738 (PMC10318366; doi:10.1016/j.patter.2023.100738)
Supplement: Document S2. Article plus supplemental information [file mmc2.pdf]

Article

Patterns

Network embedding unveils the hidden interactions in the mammalian virome

Graphical abstract

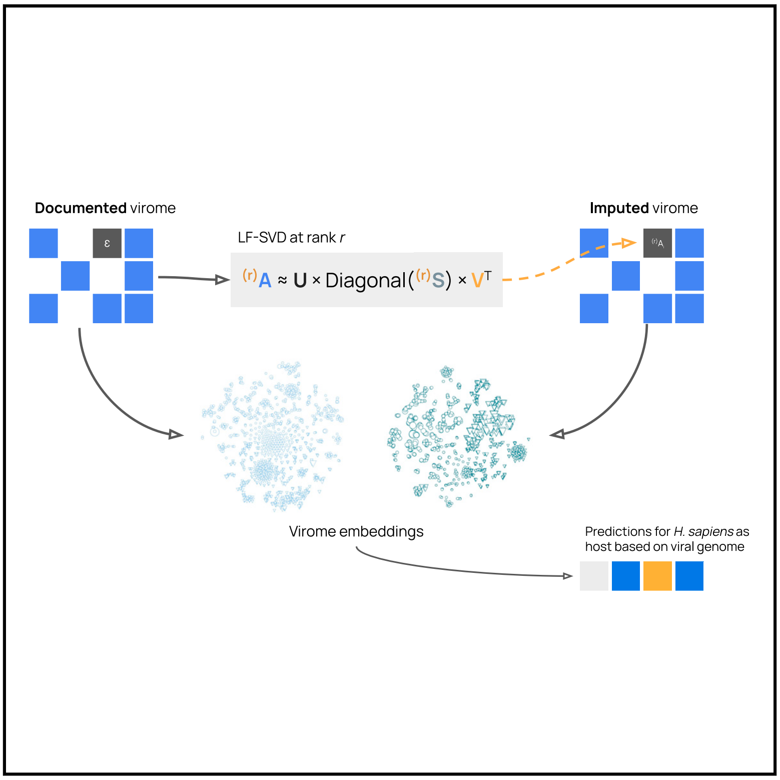

Authors

Timothée Poisot,
Marie-Andrée Ouellet,
Nardus Mollentze, ..., Rory J. Gibb,
Stephanie N. Seifert, Colin J. Carlson

Correspondence

timothee.poisot@umontreal.ca

In brief

Understanding host-virus interactions is critical to better anticipate where the next pandemic may come from. In this article, we build a new recommender system that is able to infer missing host-mammal interactions. We show that it solves well-known biases in data collection while boosting the predictive ability of a model that identifies potentially zoonotic viruses.

Highlights

- A novel recommender system using network features predicts mammal-virus interactions
- Mapping these predictions in space identifies hotspots of undiscovered viral interactions
- The Amazon has undiscovered interactions, and Africa has undiscovered zoonotic hosts
- The embedded virome boosts our ability to identify potentially zoonotic viruses

Poisot et al., 2023, Patterns *4*, 100738
June 9, 2023 © 2023 The Author(s).
https://doi.org/10.1016/j.patter.2023.100738

CellPress

Patterns

CellPress
OPEN ACCESS

Article

Network embedding unveils the hidden interactions in the mammalian virome

Timothée Poisot,[1,11,*] Marie-Andrée Ouellet,[1] Nardus Mollentze,[2,3] Maxwell J. Farrell,[4] Daniel J. Becker,[5] Liam Brierley,[6] Gregory F. Albery,[7] Rory J. Gibb,[8] Stephanie N. Seifert,[9] and Colin J. Carlson[10]

[1]Département de Sciences Biologiques, Université de Montréal, Montréal, QC, Canada
[2]School of Biodiversity, One Health and Veterinary Medicine, University of Glasgow, Glasgow, UK
[3]MRC – University of Glasgow Centre for Virus Research, Glasgow, UK
[4]Department of Ecology and Evolutionary Biology, University of Toronto, Toronto, ON, Canada
[5]Department of Biology, University of Oklahoma, Norman, OK, USA
[6]Department of Health Data Science, University of Liverpool, Liverpool, UK
[7]Department of Biology, Georgetown University, Washington, DC, USA
[8]Center for Biodiversity & Environment Research, University College, London, UK
[9]Paul G. Allen School for Global Health, Washington State University, Pullman, WA, USA
[10]Center for Global Health Science and Security, Georgetown University, Washington, DC, USA
[11]Lead contact
*Correspondence: timothee.poisot@umontreal.ca
https://doi.org/10.1016/j.patter.2023.100738

THE BIGGER PICTURE Documenting all interactions between viruses and mammals is not feasible; viruses are too small, the world is too big, and viruses and mammals are too diverse. As a consequence, we think we only know about 1% or 2% of the interactions between mammals and viruses. This is a critical gap in our knowledge because it can lead us to missing reservoirs of possible zoonotic viruses. In this article, we develop a process to leverage the information we have about interactions between hosts and viruses to do three things: First, we predict missing interactions in this network and give them a score based on how likely the model guesses they are. Second, we map these predicted interactions in space to provide guidance about where to go and what to look for to collect data that would maximize our knowledge of host-virus interactions. Finally, based on the predicted interactions, we use information about the genome of viruses to identify possible zoonotic viruses.

1 2 3 4 5 **Development/Pre-production:** Data science output has been rolled out/validated across multiple domains/problems

SUMMARY

Predicting host-virus interactions is fundamentally a network science problem. We develop a method for bipartite network prediction that combines a recommender system (linear filtering) with an imputation algorithm based on low-rank graph embedding. We test this method by applying it to a global database of mammal-virus interactions and thus show that it makes biologically plausible predictions that are robust to data biases. We find that the mammalian virome is under-characterized anywhere in the world. We suggest that future virus discovery efforts could prioritize the Amazon Basin (for its unique coevolutionary assemblages) and sub-Saharan Africa (for its poorly characterized zoonotic reservoirs). Graph embedding of the imputed network improves predictions of human infection from viral genome features, providing a shortlist of priorities for laboratory studies and surveillance. Overall, our study indicates that the global structure of the mammal-virus network contains a large amount of information that is recoverable, and this provides new insights into fundamental biology and disease emergence.

This is an open access article under the CC BY license (http://creativecommons.org/licenses/by/4.0/).

INTRODUCTION

Despite growing interest in viral ecology, data remain limited because most of the global virome remains undocumented. Computational methods that can infer undiscovered associations in a partially observed host-virus network can fill in some of these gaps.[1] At least 20%–40% of host-parasite associations are estimated to be unrecorded in locally collected, highly complete datasets;[2] a much higher proportion is likely unrecorded in the high-sparsity datasets cataloging the global virome. An even greater proportion of host-virus interactions may be biologically plausible (i.e., a virus might have the capacity to infect a host) but still unrealized for lack of ecological opportunities. These are often the links with the greatest relevance to actionable science; at least 10,000 mammalian viruses likely have the unrealized capacity to infect human hosts,[3] while an even greater number could be shared thousands of times between mammals as they track shifting habitats in a changing climate.[4]

Here, we propose a novel method for predicting unknown links in partially sampled networks and apply it to the largest database of host-virus associations currently available. The method is based on a combination of linear filtering, which uses high-level network information to generate an initial guess as to the probability of an interaction, and singular value decomposition, which uses the structure of a low-rank approximation (which has a better signal-to-noise ratio[5]) of the entire network to impute interactions that were presumed negatives. In combination, this method uses existing knowledge on the entire network but can also be tuned in such a way that its adjacency matrix is approximated at a rank that maximizes the amount of information used for imputation. Importantly, this method relies entirely on network structure and does not consider (or require) external information specific to the hosts and viruses involved (Table 1). We used this method to predict host-virus associations that are either undetected (they happen in nature but are not observed or documented) or are biologically plausible but possibly unrealized in the real world (they can happen in nature but are restricted by the spatial distribution of the species, which can be modified by climate change). Finally, we applied graph embedding to the observed and imputed networks and used these as predictive features to augment a previously published model that predicts which viruses can infect humans based on summaries of viral genome composition,[6] testing whether knowledge about the global dynamics of cross-species transmission is informative for the narrowly defined problem of predicting human disease emergence.

RESULTS AND DISCUSSION

Predicting the host-virus network

The combined linear filtering and singular value decomposition (LF-SVD) model relies on four hyper-parameters describing the relative importance of network structure and matrix rank used for approximation (SVD). The network structure parameters we use are the in-degree (proportional number of viruses infecting a host), out-degree (proportional number of hosts infected by a virus), and connectance (proportion of pairs of species that have an interaction); these were picked because they capture a lot of relevant information on network structure and have been established to be enough to start identifying possible missing interactions.[7] After tuning of the hyper-parameters, the best model (which used initial values emphasizing network connectance and performed SVD at rank 12) achieved an area under the receiver operating characteristic curve (ROC-AUC) of 0.84 (Table S1; Figure S1). Although analyses of ecological networks usually gravitate toward using degree-based (over connectance-based) models, this choice of best model is perhaps unsurprising. Assuming that the overwhelming majority of interactions are unsampled, known degree is mostly a proxy for sampling effort—an assumption that is supported by previous work suggesting that observed per-host viral richness (equivalent to degree in a bipartite network) is largely the result of virus discovery effort.[8] The hyper-parameter tuning strategy and the one-by-one LF-SVD imputation step help circumvent this bias; in less under-sampled networks, or in networks where undersampling has less statistical structure, it would not be surprising to see degree-based models outperforming connectance-based ones.

We applied four tests of whether model performance was undermined by biases in the partially observed network, a common problem in predicting host-pathogen interactions.

First, we tested the effect of passive sampling bias with a regression of host species' viral diversity against citation counts, a commonly used proxy for scientific research effort. We found that, consistently, citations had a weaker effect predicting viral richness after imputation (Table S2), suggesting a direct debiasing effect.

Second, we tested the influence of impact bias, a specific form of active sampling bias driven by relevance to human health. As a simple test of impact bias, we examined the top 10 hosts that shared viruses with humans before and after imputation. In the observed network, domesticated and lab animals dominated this list; although proximity to humans might lead livestock to share many pathogens, this result is generally presumed to be the effect of impact bias. After imputation, many of these species were replaced with a handful of great apes and rodents (Figure S2). The former reflects well-supported biological rules (closely related species share more viruses[1,9]), while the latter might reflect a mix of true rodent "hyper-reservoir" potential[10] and, more likely, residual sampling bias from the well-characterized viromes of mouse and rat models.

Third, we examined whether sampling bias might be creating an undesirable "rich-get-richer" effect, where novel interactions are disproportionately predicted for species that are already oversampled. If training data were unbiased, then this could be a useful property; for example, a model might correctly assign more interactions to some viruses because it "learns" that they have a higher intrinsic host plasticity. However, host-virus networks are heavily shaped by sampling history, creating geographic and taxonomic biases that could produce false inferences.[8,11]

To examine the effects of geographic sampling bias, we mapped the number of total known host-virus interactions based on mammal host ranges and compared this with the same map of newly predicted interactions (Figure 1). In previous studies, predictive models have often reproduced a pattern of disproportionate sampling in European wildlife.[10,12] Our model suffered from a similar limitation and predicted notably fewer interactions

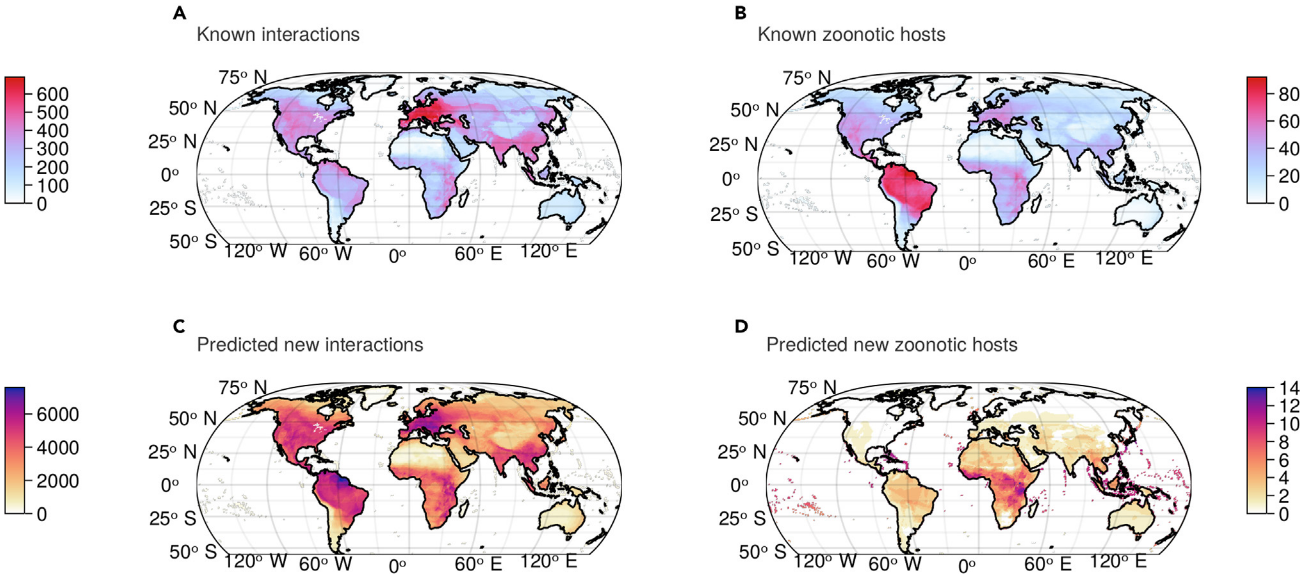

Figure 1. Mammal biodiversity and sampling bias shape the geography of predicted interactions

(A) The total number of interactions recorded does not track the global distribution of mammalian richness with an overwhelming density of interactions in Europe.

(B) Known zoonotic hosts are concentrated in the Amazon, an area with comparatively fewer known host-virus interactions; the distribution of known zoonotic hosts closely tracks the global richness of mammals.

(C and D) Post imputation, the model predicts strong increases in the number of interactions (C) in the Amazon and Central Europe but an increase in the number of zoonotic hosts primarily concentrated in Africa (D). As a result, we expect the Amazon to be a hotspot of novel interactions and Africa to be a hotspot of novel zoonotic hosts (i.e., the increase is greater than expected, given the known quantities in these places).

in South America and Africa. However, the imputation process did significantly reduce geographic bias, and the imputed interactions tracked global gradients in mammal biodiversity much better than the original network. We also repeated this analysis just for mammalian hosts of zoonotic diseases and found that the original network was heavily biased toward neotropical rainforests but predicted that zoonotic hosts were primarily concentrated in sub-Saharan Africa. This would be a notable departure from previous work, which has again reproduced patterns in existing data and predicted that undiscovered zoonoses are largely concentrated in the neotropics.[13,14]

To examine the effects of taxonomic sampling bias, we estimated the number of "missing" viruses (i.e., the gap between observed and estimated viral richness per host species[13]) by counting each host's predicted novel interactions. Missing viruses displayed only a moderate phylogenetic signal (Pagel's $\lambda = 0.35$), suggesting that they are distributed fairly equally across the mammalian tree of life—a finding that matches other recently published observations.[15] An additional taxonomic analysis identified four clades—cetaceans, a subclade of mostly insectivorous bats, and two subclades of New World rodents–with fewer missing viruses than other mammals (Figure S3; Table S3), suggesting that the model is responsive to the fact that these taxa may be more deeply sampled than the average mammal. In the last two decades especially, bats and rodents have been prioritized by virus surveillance programs because they account for the majority of mammal species diversity and because of hypotheses about their role as disproportionate "hyper-reservoirs" of zoonotic diseases (e.g., Han et al.[10] and Luis et al.[16]). A growing number of analyses suggest that these clades may not actually harbor more viruses or more zoonoses; rather, the appearance that they do is an impact of this disproportionate sampling effort.[8,15,17,18] Our model assigns fewer unknown interactions to these species, suggesting that it has the ability to overcome taxonomic sampling bias without directly using any data on host taxonomy or phylogeny.

Emergent properties of the imputed host-virus network

Compared with the 5,494 interactions recorded in our original mammal-virus dataset, our model predicted a total of 75,901 new interactions (Figure 2). With a total of 81,395 interactions, the imputed network has a connectance of 0.09, which is well within the range of connectances for antagonistic bipartite networks.[19] The best-scoring model has a false discovery rate of 9.3%, meaning that it is potentially over-predicting about 7,060 interactions. The same model has a false omission rate of 23%, which would suggest a number of undiscovered interactions of the order of 10^5 for this dataset. This being said, these numbers should be interpreted within the context of data constraints; the initial dataset is biased toward extreme sparsity, and for this reason it is likely that the imputed network is less severely incomplete than the false omission rate would suggest.

We next examined the post-imputation network for meaningful biological signals. The "evolutionary distance effect" is often the best-supported signal in host-virus networks: closely related hosts share viruses (through coevolution) and microbiologically relevant traits (through identity by descent), which facilitates cross-species transmission, leading to a correlation between evolutionary distance and virome similarity.[9] We tested this property in the pre- and post-imputation networks by examining viral sharing pairwise among all hosts and between humans and other mammals. We found a strong and consistent phylogenetic

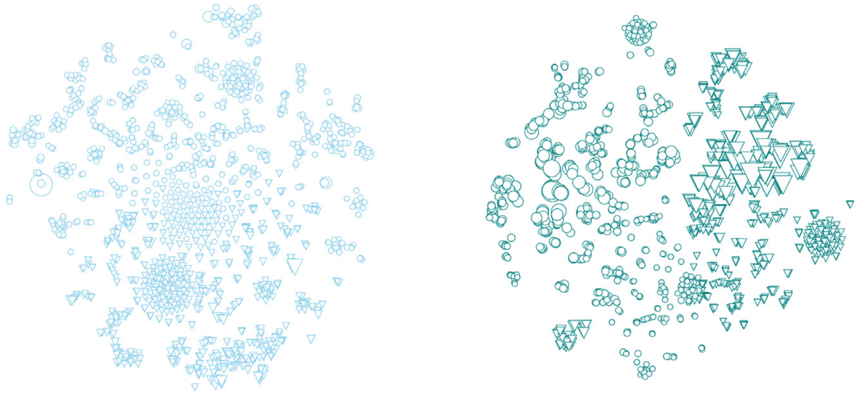

Figure 2. The global virome pre and post imputation

Network layouts reflect the first two dimensions of a t-SNE embedding on four dimensions, where the positions of nodes were initially picked based on a PCA analysis. Hosts are shown as circles and viruses as downward-pointing triangles, and the relative size of each point scales linearly with degree (using the same scale for both figures; i.e., two nodes with the same degree will have the same size on the left and right).

distance effect in viral sharing (whether two hosts share any viruses at all) and the total number of viruses shared pairwise among mammals and specifically with humans (Figure S4); although imputation reduced the signal of these effects, all but one (binary viral sharing with humans) remained significant even after imputation (Tables 2 and 3). These results suggest that the interactions predicted by our model have a high biological plausibility, and that, even without incorporating any host or viral traits into our analyses, the latent factors that structure the network are identified and successfully recapitulated by the model.

Finally, we evaluated the effect of imputation on the spatial distribution of viral biodiversity. Our models predicted that the total number of host-virus interactions generally tracked mammal biodiversity, with a previously unknown hotspot of potential zoonotic disease hosts in sub-Saharan Africa (Figure 1). To further explore these patterns, we used the local contribution to the beta-diversity approach,[25] which measures the extent to which the community at a single location differs from the expectation based on the entire range considered. When applied to interactions,[26] it reveals areas where, although the network might not be structurally different, it is composed of interactions that do not usually occur together. In biological terms, this means that novel host jumps are possible through different host-virus pairs being in contact. Comparing the uniqueness of the viral community composition based on host spatial distribution before and after imputation reveals an undocumented hotspot of unique host-virus associations in the Amazon (Figure 3). This finding tracks with other recent work on the biogeography of bat coronaviruses,[27,28] which has suggested that betacoronaviruses followed divergent trajectories of cospeciation with their hosts after some bat families became isolated in the New World. Our predictions suggest that this might be a broader pattern that shaped the biogeography of mammal viruses, and although the Amazon may not harbor disproportionate viral richness, it might be home to more unusual (and currently unknown) branches of viral evolution.

Table 1. Comparison with existing approaches

	Network	Taxonomy	Phylogeny	Traits	Sampling effort
This study	yes				
Stock et al.[7]	yes				
Evans et al.[20]		host, virus		host, virus	
Farrell et al.[12]	yes		host		
Pandit et al.[21]	yes	virus			yes
Stock et al.[22] *	yes	virus	host	host	yes
Wardeh et al.[23]	yes	virus	host	host, virus	yes

Our study is one of the only feature-agnostic approaches that has so far been applied to predicting mammal-virus interactions and results in equally biologically plausible findings compared with other approaches. These previous approaches usually require extensive information on host and virus features, which often requires an estimate of sampling effort to be added as a confounder. By adding the SVD step after the LF, we improve on the Stock et al.[7] approach, which is equally feature agnostic but focuses on the network rather than its embedding; this method performed poorly when applied to prediction of bat hosts of betacoronaviruses in a multi-model context,[24] emphasizing the importance of considering network embeddings even lacking additional predictors. Some of the methods (marked with asterisks) listed have only been applied to non-virus systems but are classified with the host-analog and virus-analog organism.

Predicting viruses with zoonotic potential

We finally explored whether network-wide prediction offered useful insights into zoonotic potential, the ability of a virus to infect humans (a subset of links with one focal node in the network). Surprisingly, we found that the imputation method did not predict known human-associated viruses any better than random (AUC = 0.51; Table 4). This finding does reassuringly imply that zoonotic viruses are not contributing a particularly strong structural bias to the predictions but indicates that the model performs poorly when predictions are restricted to one fairly atypical node of over 1,000. Indeed, while the ability of the model to predict the viruses associated with a given host generally increased as hosts are linked to more viruses, performance was poor for hosts linked to unusually high numbers of viruses relative to the rest of the dataset (of which humans were the most extreme; Figure 4). A similar but less extreme pattern was observed among viruses linked to above-average numbers of hosts. Thus, although our best model focusing exclusively on connectance performed well in general, models incorporating in- or out-degree or specialized to a particular node may be needed for better-sampled nodes.

CellPress
OPEN ACCESS

Table 2. Phylogenetic signal in virus sharing pre and post imputation

Sharing	Data source	β	SE	p Value	R^2 (adj.)
Pairwise (all hosts)	pre imputation	2.23 e−2	8.44 e−05	***	9.8%
Pairwise (all hosts)	post imputation	3.50 e−03	1.30 e−4	***	0.07%
With *H. sapiens*	pre imputation	3.32 e−2	1.07 e−2	***	2.4%
With *H. sapiens*	post imputation	1.46 e−2	2.30 e−2	0.524	−0.09%

Statistics are given for a GLM fit with a binomial distribution for the outcome variable (whether any viruses at all are shared between two hosts). ***$p < 0.001$.

Table 3. Phylogenetic signal in number of viruses shared pre and post imputation

Sharing	Data source	β	SE	p Value	R^2 (adj.)
Pairwise (all hosts)	pre imputation	−2.04 e−02	4.97 e−05	***	8.5%
Pairwise (all hosts)	post imputation	−6.12 e−03	7.20 e−06	***	3.0%
With *H. sapiens*	pre imputation	6.53 e−03	4.50 e−04	***	2.2%
With *H. sapiens*	post imputation	4.76 e−0.3	1.08 e−04	***	3.8%

Statistics are given for a GLM fit with a Poisson distribution for the outcome variable. ***$p < 0.001$.

We next investigated whether the imputed host-virus network could be applied in specialized models aimed at identifying human-infecting viruses. Viral host breadth is a widely used predictor of zoonotic ability but is generally unavailable for poorly studied viruses.[6,13,29] To test whether the structural information on host range from our imputed network can be made accessible for prediction, we revisited a recently developed model that applies boosted regression tree models to predict zoonotic potential based on the genome composition of animal viruses.[6] We extracted the position of viruses in the pre- and post-imputation networks by removing humans (as well as viruses linked only to humans in the observed data) and applying random dot product graph embedding, which generated a total of 12 latent features that describe each virus's relationship to other viruses and animal hosts in the network. We then added these features to the genome composition-based model and compared performance on the same set of viruses. Models incorporating the embeddings performed significantly better than a genome composition-only model despite the fact that humans were removed from the network. Using embeddings derived from the post-imputation network consistently produced better predictions (mean test set AUC = 0.875, SD = 0.04; Figure 5). Averaging predictions across the top 10% of repeated training iterations[30] further improved performance (AUC = 0.898). Moreover, of the top 20 viruses predicted by the algorithm, 11 already have serological or otherwise circumstantial evidence of human infection (Table 5), as do many of the other highly ranked viruses (Figure 6).

Overall, these findings suggest that network inference and network embedding can work in tandem to capture latent information about viral ecology and evolution, leading to better predictions about which viruses might someday infect humans. However, more work is needed to establish the exact operating conditions under which such an approach can be useful; in particular, the number of animal hosts that need to have been found before reliable inferences on zoonotic risk can be made for novel viruses (cf. Figure 4) is difficult to assess without detailed data on the order in which hosts are linked to viruses (expected to be nonrandom given sampling biases).

Conclusions

In this study, we use a novel feature-agnostic imputation method to infer properties of the host-virus network that are often clouded by sampling bias or data deficiency. Our findings support the general assumption that only a small percentage of the mammalian virome has been characterized, even in well-sampled regions like Europe. However, our models also suggest some new aspects of global viral biogeography; in particular, we find that future virus discovery efforts in the Amazon may reveal a hidden hotspot of unique coevolutionary systems, while future sampling in sub-Saharan Africa might be most likely to identify new reservoirs of zoonotic disease. Applying the model to zoonotic risk ranking of wildlife viruses, we find that ecological networks contain a substantial amount of information that can be recovered through graph embedding and machine learning. Our shortlist of predicted high-risk viruses could be a starting point not just for laboratory characterization but for real-world surveillance, especially for pathogens where we found some evidence that emergence in human populations may already be underway. Future work can expand these findings by adding more microbiological, immunological, and ecological mechanisms,[1] eventually iterating a living model of the global virome.

Our study provides a strong proof of concept that the structure of the observed host-virus network contains meaningful information about the rules of cross-species transmission. The imputation process recovers more of this information, even without use of mechanistic predictors like host phylogeny, retaining biologically relevant signals while reducing key biases in current observational data. Thus, future efforts to predict viral emergence may be able to leverage use of recommender systems as a data inflation step to make better predictions. However, these approaches (and notably their validation) remain limited by how poorly characterized the host range of most viruses is; the majority of viruses are either undiscovered or known from a single host. As the global virome becomes better sampled, these approaches will be increasingly reliable not just for biological inference but for actionable efforts to prevent zoonotic emergence.

EXPERIMENTAL PROCEDURES

Resource availability

Lead contact

The lead contact for this work is Timothée Poisot (timothee.poisot@umontreal.ca).

Materials availability

Does not apply.

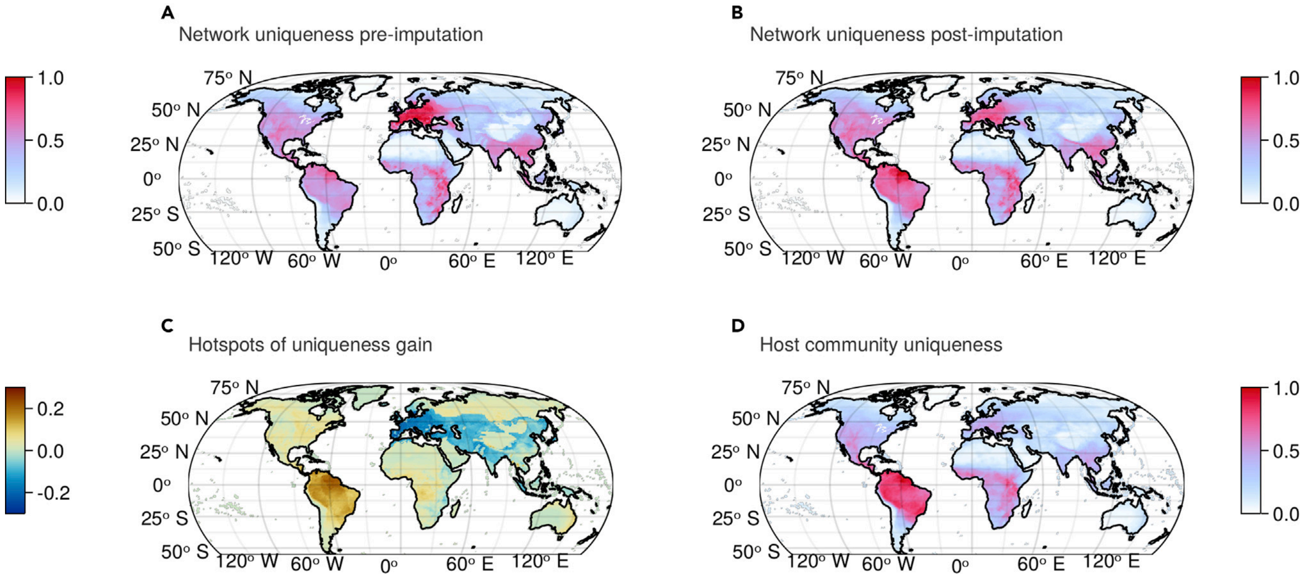

Figure 3. Network imputation reveals a hotspot of unique host-virus associations in the Amazon
(A and B) The compositional uniqueness of host-virus interactions remains about similarly distributed in the pre-imputation (A) and post-imputation (B) networks.
(C) Nevertheless, the largest hotspot in gain of interaction uniqueness is in the Amazon.
(D) It appears that the predicted hotspots of uniqueness gain closely follow the originality of the host compositions, suggesting that more unique mammal assemblages have more original host-virus networks. Hotspots are given as the difference in uniqueness post and pre imputation, both rescaled between 0 and 1.

Data and code availability

The code to reproduce these analyses is accessible online under the MIT license (https://github.com/viralemergence/trefle[39]); the code for zoonotic virus imputations is similarly available online under the GPL license (https://github.com/viralemergence/haystack[40]).

Model design and implementation

Host-virus association data

We used a recently published dataset called CLOVER,[41] which is the largest open dataset describing the mammal-virus network currently available and combines data from four sources that each cover overlapping but distinct portions: the Host-Pathogen Phylogeny Project (HP3) dataset,[13] the Enhanced Infectious Diseases Database (EID2),[42] the Global Mammal Parasite Database version 2.0 (GMPD2),[43] and an unnamed dataset recently published by Shaw et al.[44] By reconciling these datasets and their underlying taxonomy, the CLOVER dataset achieves a 30% reduction in matrix sparsity over the next most detailed dataset.

The CLOVER dataset describes 5,494 interactions between 829 viruses and 1,081 mammalian hosts. The majority of these interactions have been recorded in wild animals using a combination of detection methods (usually serology, PCR, or virus isolation). A small portion of records assimilated from NCBI's GenBank into these other datasets may also record experimental infections, which provides insight into biological compatibility but not necessarily opportunity for infection in nature. Each of the component datasets and the CLOVER dataset are presence only (i.e., they only report an edgelist of known interactions and do not include true negatives).

Imputation model description

The imputation model uses two steps to chain LF (which can recommend potentially false-negative interactions[7]) to recommendation based on SVD (which adequately captures the low-rank structure of ecological association networks[45]). This imputation model is hereafter termed LF-SVD. The LF step relies on four hyper-parameters expressed as an array of weights $\alpha = [\alpha_1, \alpha_2, \alpha_3, \alpha_4]^T$, which are, respectively, the relative importance of the original (i.e., observed) value of the interaction, in- and out-degree, and connectance

Table 4. The top 10 predicted (novel) zoonotic links in the post-imputation network

Virus	Family	Evidence	Prior risk assignment
Canine mastadenovirus A	Adenoviridae	275.6808	medium
Simian mastadenovirus A	Adenoviridae	242.8597	–
Panine gammaherpesvirus 1	Herpesviridae	201.9715	–
Phocid alphaherpesvirus 1	Herpesviridae	191.4652	high
Carnivore protoparvovirus 1	Parvoviridae	191.2557	high
Torque teno virus 14	Annelloviridae	187.3940	high
Torque teno virus 4	Annelloviridae	187.3940	medium
Panine betaherpesvirus 2	Herpesviridae	187.3940	high
Torque teno virus 23	Annelloviridae	187.3940	high
Torque teno virus 2	Annelloviridae	182.4210	medium

Evidence of interaction generated by the imputation model is contrasted against prior predictions by Mollentze et al.,[6] who implemented a model that successfully predicts zoonotic potential from viral genome composition bias. Revised estimates of this model applied to the imputed network are presented in Table 5.

CellPress
OPEN ACCESS

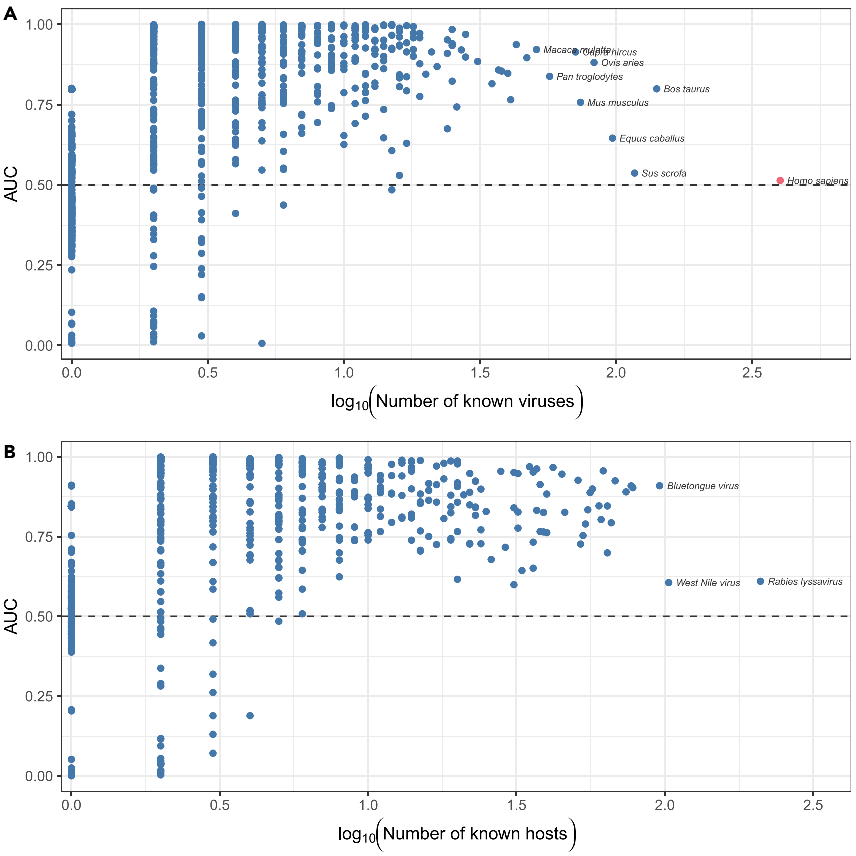

Figure 4. Predictive performance of LF-SVD generally increases with increased connectivity

Points represent individual host species and show the probability that a randomly sampled virus known to infect that host will be ranked above a randomly sampled virus that has not been observed to do so (measured as the area under the receiver operating characteristic curve [ROC-AUC]). While hosts subject to extreme study bias, such as humans, cannot be predicted, this does not appear to degrade performance on other species.

(the constraint $\sum \alpha = 1$ is always enforced). LF creates a potential matrix $\mathbf{A}$ from an observed matrix $\mathbf{Y}$ of size (n, m) by assigning every interaction between species i and j an initial score given by the dot product of weights and properties of $\mathbf{Y}$,

$$A_{ij} = \left[Y_{ij}, \frac{1}{n}\sum_k Y_{kj}, \frac{1}{m}\sum_l Y_{il}, \frac{1}{nm}\sum \mathbf{Y}\right] \cdot \alpha.$$

This corresponds to a weighted average of averages, wherein $\forall (i,j), 0 \leq A_{ij} \leq 1$. We compared three parameterizations of the α: connectance only ($[0,0,0,1]^T$), degree only ($[0,1,1,0]^T$), and hybrid ($[0,1,1,1]^T$). While technically there is an infinite number of possible configurations for the LF weight vector, the computational cost of a grid search is prohibitive, and these parameterizations have the added benefit of corresponding to phenomenological assumptions about what drives network structure that have been well laid out in the literature.[19] In this application, we set $\alpha_1 = 0$, as the initial value of the interaction is ignored, reducing the number of hyper-parameters to tune from four to three.

We updated the initial values produced by LF using (truncated) SVD imputation. Like principal-component analysis (PCA), SVD is an embedding of a starting matrix into latent subspaces; compared with PCA, SVD is a more general solution that also well handles numerical instability because of very small entries,[46] which is a likely scenario because some interaction probabilities are expected to be small. Because all entries of $\mathbf{A}$ and $\mathbf{Y}$ are in $\mathbb{R}$, we can decompose either of these matrices as $\mathbf{U\Sigma V}^T$, where $\mathbf{U}$ and $\mathbf{V}$ are unitary matrices known as the left and right subspaces, and $\mathbf{\Sigma}$ is a diagonal matrix containing the singular values of the decomposed matrix. To impute the interaction (i,j), we create a matrix $\mathbf{K} = \mathbf{Y}$, wherein $K_{ij} = A_{ij}$ (according to the LF model). To decompose this matrix at low-rank r, we set the values of $\mathbf{\Sigma}$ larger than k to 0 and calculate the approximate version of $\mathbf{K}$ as

$$\overline{\mathbf{K}} = \mathbf{U}_r \mathbf{\Sigma}_r \mathbf{V}_k^T$$

The overall SVD step was conducted as follows: for every interaction (i, j), we first set its value according to the LF model and perform the truncated SVD step as outlined above. We then update $\mathbf{K}$ so that $K_{ij} = \overline{K}_{ij}$. The SVD step is repeated 20 times (after preliminary assays revealed that the absolute change after 10 iterations was consistently smaller than 10^{-3}), and the final value after 20 iterations is the score for the imputed interaction. Note that, because of the nature of SVD, the score is not bound to $[0,1]$; for this reason, we re-expressed the score as "evidence of increase" (measured as the ratio between the updated and initial value minus one so that an initial score that is unaffected by SVD has a value of 0), and brought this value back to the unit interval by taking its logistic. This yields a pseudo-probability for the interaction, which is then thresholded (during hyper-parameter tuning) and used for imputation. The tuning and imputation of the LF-SVD model were performed in the software Julia 1.6[47] using the `EcologicalNetworks.jl` package.[48]

Hyper-parameter tuning, thresholding, and evidence scoring

To tune the hyper-parameters (LF weight vector, SVD rank), we picked a calibration set of 800 positives and 800 assumed negative interactions and imputed them using each possible model (using ranks from 1–20, giving $n = 60$). This makes the strong assumption that the 800 negative interactions we picked in the calibration set were indeed true negatives; although the model ended up recommending many interactions, ecological networks are known for their sparsity, and we judged this assumption acceptable based on an overall examination of model performance.

Outside the field of host-virus network prediction, our approach allows us to establish a data-driven baseline for the seeding of SVD as a recommender. This is an important development because we show a flexible method to account for different aspects of network structure; although, in this instance, the best possible tuning used connectance as an initial values, networks with different degrees of undersampling may be best predicted by initializing the recommender step with values derived from, e.g., their degree distribution.

For each set of 1,600 predictions returned by the models, we derived confusion tables at thresholds ranging from the lowest to the highest score using 1,000 steps; recall that the thresholding is performed on the transformed score on the unit interval so that the step size is constant ($\approx 10^{-3}$). From this confusion table, we calculated the ROC-AUC, true/false positive/negative rates, positive/negative predictive values, false discovery/omission rates, critical success index, accuracy, and informedness (also known as Youden's J). The model with the highest ROC-AUC was picked as the best model and used for the rest of this study.

The exact cutoff to use to transform the continuous output of LF-SVD into a binary classifier (i.e., the interaction is recommended or not) was determined by picking the threshold value maximizing Youden's J statistic. Each interaction is presented as an evidence score, which is obtained by dividing the values post-imputation (LF-SVD) by the values pre-imputation (LF), minus

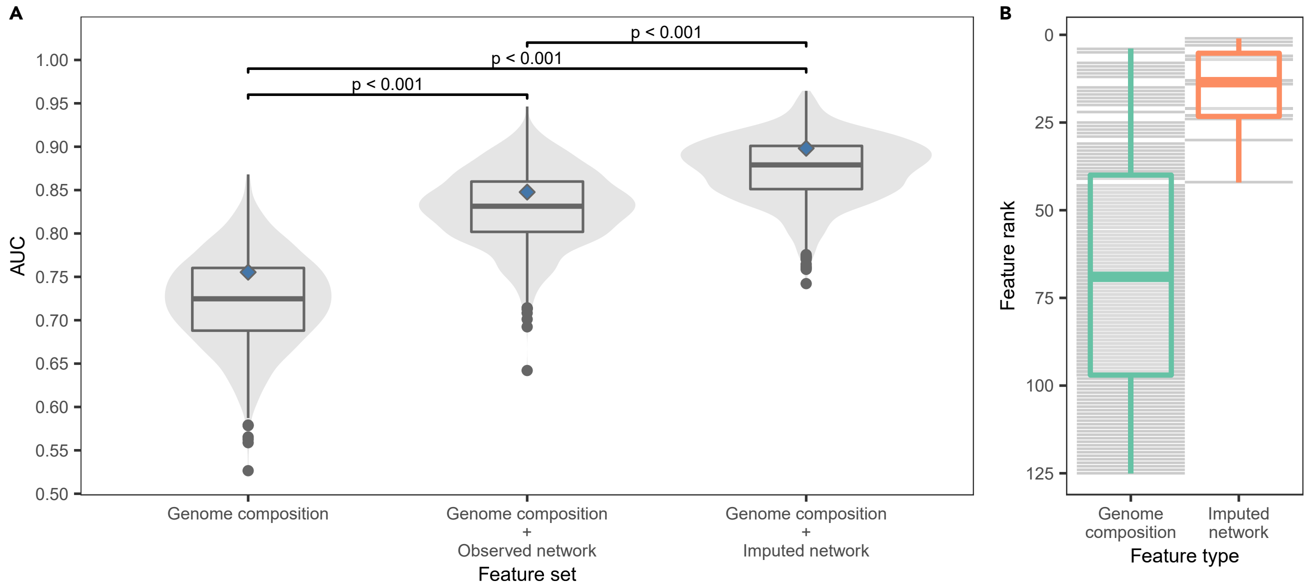

Figure 5. Network embeddings improved the ability to identify viruses that can infect humans

(A) An existing model of human infection risk using virus genomic features is improved when network embeddings are added as virus traits; models that use embeddings from the imputed network perform better than those using the observed network. Violin and boxplots show the ROC-AUC for test set predictions across 1,000 replicate 70%:15%:15% train:calibrate:test splits (n = 612). p-values from pairwise Kruskal-Wallis rank-sum tests are shown for all comparisons. Diamonds indicate the performance of a bagged model that averages predictions from the 100 best-performing models based on test set AUC iteratively re-calculated while excluding the virus being predicted. Mean AUC: genome composition model = 0.723; genome composition + observed network = 0.830; genome composition + imputed network = 0.875.

(B) Predictive feature importance in the combined (genome composition + imputed network) model; network embeddings are consistently the top predictive features compared with biologically informative measures of genome composition.

one. An evidence of 0 means that the imputation did not change the value, and increasingly positive values meant that the change because of imputation was stronger. This interaction evidence was used to rank interactions when required for the analyses.

Comparison to existing approaches

The LF-SVD method is fairly unique as a feature-agnostic method to predict bipartite ecological networks. Previous studies that have developed predictive models of the mammal-virus network have generally included a mix of host and viral traits as predictors (Table S1), generally using a machine learning classifier such as boosted regression trees. Our method uses a network theoretic approach at the global scale rather than assigning node-level features to include in a standalone machine learning model and is entirely agnostic to host and virus traits. Our approach is most comparable with a handful of approaches that use network dissimilarity to structure recommendations, sometimes alongside host phylogeny[12] and other traits.[22] A growing number of comparable studies also leverage network features like network motifs[23] or other topological metrics[21] at the node level and add these to a base machine learning approach that is network agnostic. Particularly in comparison with a previous standalone iteration of the LF-only approach,[7] which validates poorly compared with ecological models,[24] our approach is surprisingly comparable with these more intensive algorithms; as we discuss, our predictions have a high degree of biological plausibility and handle most kinds of sampling bias well, including some like impact bias and rich-gets-richer effects that are particularly visible in previously published work.[12,23]

Analysis of the imputed network

Additional data sources

For phylogenetic analyses, we used a recently published mammalian supertree published by Upham et al.[49] that has been taxonomically harmonized to the CLOVER dataset for ease of analysis. For geographic analyses, we used the International Union for Conservation of Nature (IUCN) Red List (iucnredlist.org) species distribution maps for mammals, downloaded on June 6, 2019. For citation counts, we extracted total virus-related publications for each species (by searching for host species binomial plus all known synonyms and "virus" or "viral") from the PubMed database using the R package `rentrez`.[50]

Testing effects of biased data collection

Observed host-pathogen association networks compiled from published records are influenced by a passive sampling bias resulting from differential research across host and pathogen species. In comparative analyses of viral richness per host species, the number of publications per host species is often included as a covariate in an attempt to control for variable sampling effort across hosts.[51] This estimate of sampling bias is consistently positively related to viral richness and typically is the strongest predictor, explaining more variation than other biological covariates.[13,16,52–54] To explore whether network imputation via LF-SVD is extrapolating sampling biases across host species, we conducted a set of phylogenetic regressions of the relationship between viral richness and the number of publications per host species (in total and limited to those including keywords about viruses). Models were fit using the formulation of phylogenetic least-squares regression provided via the `pgls` function (Pagel's λ estimated via maximum likelihood) in the R package `caper`.[55,56] By comparing models of observed viral richness with estimates after imputation with LF-SVD, we investigate the slope of the relationship and the explained variance in viral richness to assess how strongly passive sampling biases are retained in the LF-SVD imputed network.

In addition to passive sampling bias, host-virus association data are frequently shaped by active or impact bias, where surveillance is targeted based on relevance to human health or economics. This is easily detected in records of virus sharing with humans. In principle, the species with the highest similarity to the human virome should be species that are closely related to humans (primates) or frequently live alongside humans (domesticated animals or synanthropic wildlife, particularly rodents that can live in human settlements), but domesticated animals and laboratory model systems will also score disproportionately in this metric because of sampling effort. As a new test of model bias, we propose that imputation should reduce the signal of the latter group in viral sharing with *Homo sapiens*, leaving mostly the former. To test the

Table 5. The top 20 predicted (novel) zoonotic viruses in the extended model

Virus	Virus family (-viridae)	Animal hosts (number of species)	Probability	Prior risk
Lagos bat lyssavirus[a]	Rhabdo	Chiroptera (10), Carnivora (3), Rodentia (1)	0.856	very high
Tacaribe mammarenavirus[b]	Arena	Chiroptera (9), Rodentia (1)	0.793	high
Rio Bravo virus[b]	Flavi	Chiroptera (19)	0.779	medium
Dera Ghazi Khan orthonairovirus[b]	Nairo	Rodentia (4), Artiodactyla (2)	0.755	medium
Wad Medani virus	Reo	Artiodactyla (6), Rodentia (4)	0.750	medium
Enterovirus E[c]	Picorna	Artiodactyla (1), Primates (1)	0.745	low
Phocine morbillivirus	Paramyxo	Carnivora (22)	0.741	high
Bimiti orthobunyavirus[b,d]	Peribunya	Chiroptera (5), Rodentia (4), Perissodactyla (1)	0.734	high
Bujaru phlebovirus[b,d]	Phenui	*P. guyannensis* (Rodentia)	0.733	very high
Ectromelia virus[e]	Pox	Rodentia (3), Carnivora (1)	0.701	high
Murine respirovirus[f]	Paramyxo	Rodentia (9), Artiodactyla (1), Carnivora (1), Primates (1)	0.683	medium
Akabane orthobunyavirus[g]	Peribunya	Artiodactyla (31), Perissodactyla (4), Proboscidea (1)	0.682	high
Reston ebolavirus[b,c,h]	Filo	Chiroptera (9), Artiodactyla (1), Primates (1)	0.680	high
Saboya virus[i]	Flavi	Rodentia (4), Chiroptera (1)	0.679	high
Simian orthorubulavirus[b,c]	Paramyxo	*M. fascicularis* (primates)	0.678	high
Chobar Gorge virus[b,d]	Reo	Artiodactyla (2), Chiroptera (2), Perissodactyla (1)	0.673	medium
Issyk-Kul virus[j]	Nairo	Chiroptera (13)	0.672	–
Patois orthobunyavirus[b,c]	Peribunya	Rodentia (6), Artiodactyla (2), Didelphimorphia (2), Carnivora (1), Lagomorpha (1)	0.667	high
Bovine fever ephemerovirus	Rhabdo	Artiodactyla (30), Proboscidea (1)	0.660	medium
Minatitlan orthobunyavirus	Peribunya	Primates (1), Rodentia (1)	0.654	–

All are classified as "very high" risk by the combined model, which uses viral genome compositions and imputed network embeddings. Prior risk assignments from Mollentze et al.[6] are also given where possible.
[a]Serological evidence recorded from four human samples.[32]
[b]Indicates that a virus has serological evidence of human infection in CLOVER, which was not included as a positive in the genomic model but was considered evidence of association in the mammal-virus network; however, note that *H. sapiens* and its associations were dropped before generating embeddings.
[c]Indicates that a virus is accepted as a human virus by Woolhouse and Brierley.[31]
[d]Indicates that a virus has recorded evidence of human infection in the Centers for Disease Control and Prevention (CDC) ArboCat, although original source literature is not traceable.
[e]A strain was isolated in 2012 from an outbreak of erythromelalgia-associated poxvirus in rural China in 1987;[33] most databases do not record this virus as zoonotic.
[f]Tentative serological evidence recorded.[34]
[g]Serological evidence recorded.[35]
[h]Serological evidence first recorded from cases associated with occupational exposure.[36]
[i]Serological evidence recorded for Potsikum virus,[37] now a member of Saboya virus.
[j]Tentative evidence of viral isolation is recorded.[38]

effect of active sampling bias, we examined the top 10 hosts based on similarity to *H. sapiens* pre and post imputation. Before imputation, the top 10 list (based on Jaccard similarity of host and human viral community) includes six livestock or companion animals (*Bos taurus*, *Equus caballus*, *Sus scrofa*, *Ovis aries*, *Capra hircus*, and *Canis lupus familiaris*), three primates (*Pan troglodytes*, *Macaca mulatta*, and *Macaca fascicularis*), and one synanthropic and commonly studied laboratory animal (*Mus musculus*). After imputation, four of the domesticated or primate species remained (*C. lupus familiaris*, *E. caballus*, *S. scrofa*, and *P. troglodytes*). The updated list includes two more primates (*Gorilla beringei* and *Gorilla gorilla*) and four more mice or rats (*Hylaeamys megacephalus*, *Peromyscus maniculatus*, *Proechimys guyannensis*, and *Zygodontomys brevicauda*). This mostly reflects changes in the network connectivity; all but one of these are in the top 10 species to gain links (with *Z. brevicauda* replaced by *Rattus rattus*).

Phylogeographic signals of missing interactions

The distribution of missing viruses (each host species' total number of predicted but unknown host-virus links) across space and across the evolutionary tree, are interlinked patterns that are of significant interest to viral ecologists.[13] These patterns inform scientists' understanding of where undiscovered zoonotic threats might emerge and can be used to target sampling to locations and taxa with the most undiscovered viruses. However, these predictions are also difficult to disentangle from sampling bias, which can create spurious patterns that are undermined on closer analysis.[15]

To assess phylogenetic patterns in the number of missing viruses, we used the previously specified supertree.[49] To match virus data against the phylogeny, we averaged missing virus counts for 30 species ($n = 14$ tips in the supertree). We used the `caper` R package to first broadly estimate phylogenetic signal as Pagel's λ.[57] We next applied a graph-partitioning algorithm, phylogenetic factorization, to more flexibly identify mammal clades that differ in missing virus counts. We used the `phylofactor` R package to partition counts of missing viruses in a series of generalized linear models with a negative binomial distribution.[58] We determined the number of significant clades using Holm's sequentially rejective test with a 5% family-wise error rate.

We identified a weak to moderate overall phylogenetic signal in the number of missing viruses ($\lambda = 0.35$), although this estimate was distinct from phylogenetically independent models and Brownian motion models of evolution (both $p < 0.01$). Phylogenetic factorization, in turn, identified only four small clades with significantly different counts of missing viruses, all of which had fewer

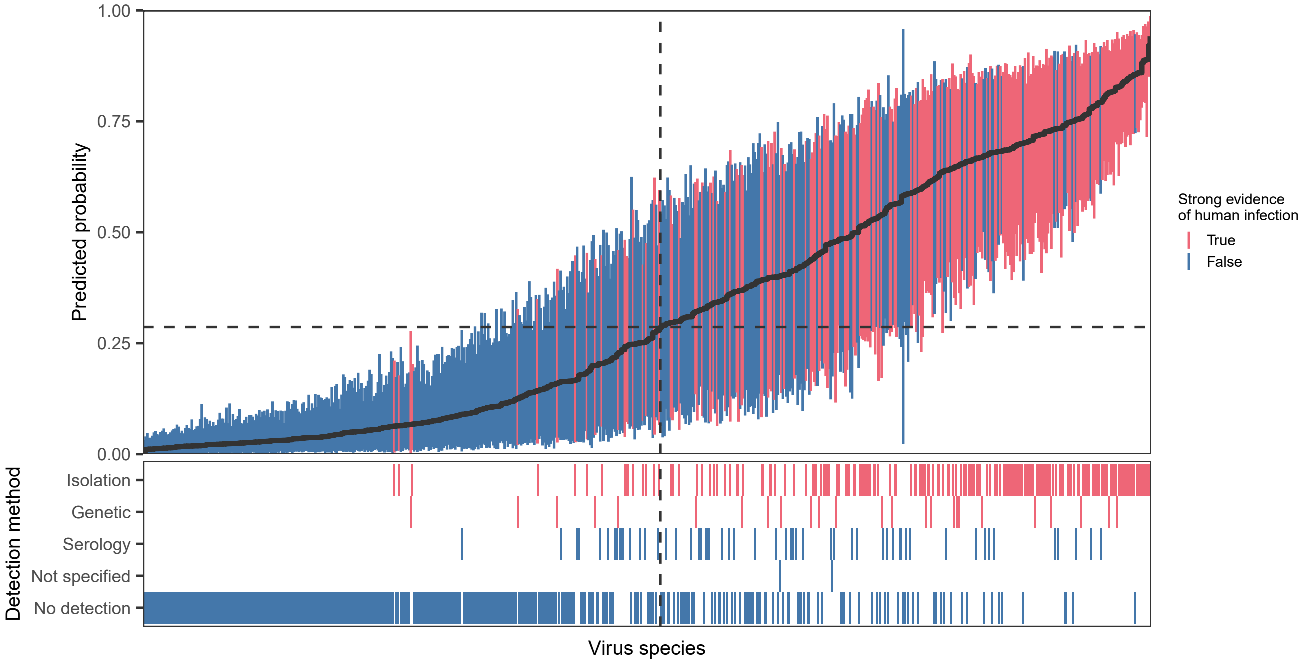

Figure 6. Ranking viruses by their predicted probability of human infection accurately predicts known infections

Viruses are arranged by the mean prediction produced by a bagged version of the model trained on genome composition features and an embedding representing the imputed network (panel A; black line). Error bars show the region containing 95% of the predictions used for bagging. Dashed lines highlight the cutoff that maximizes informedness (Youden's J) when converting mean predicted probabilities to binary predictions. Panel B shows the most reliable detection method providing evidence of human infection for each virus in the CLOVER database. For the purposes of model training, viruses linked to humans through serological detection only or where the detection method was unspecified were labeled negative; the model nevertheless identifies the majority of these as human infecting.

missing viruses than the remaining mammal phylogeny (Table S3). These clades included cetaceans ($\bar{x}$ = 18, n = 30) and a subclade of primarily insectivorous Yangochiroptera ($\bar{x}$ = 43, n = 109) as well as two subclades of the New World rodent subfamily Sigmodontinae ($\bar{x}$ = 11, n = 11; $\bar{x}$ = 16, n = 15). Overall, these results indicate that, except for some coldspots likely driven by oversampling (or, in the case of cetaceans, a peripheral role in the host-virus network), missing viruses are distributed fairly equally across the mammalian tree of life—a finding that matches other recently published work.[15]

To assess geographic patterns in the number of missing viruses, we evaluated the number of known and missing viruses at the level of each host species and joined these to each host's IUCN range map. We mapped the total number of hosts with recorded interactions, the total number of known and predicted missing interactions, and the normalized difference between missing interactions and host diversity. Known interactions are recorded disproportionately in Europe and Asia and, to a lesser degree, North America, a pattern that reveals strong sampling bias in viral inventories (Figure 1). This pattern is substantially reduced in the missing interactions, which globally track the true distribution of mammal diversity fairly well (better, in some places, than the hosts with viral interactions recorded in CLOVER). However, the normalized difference map still revealed a bias toward interactions predicted in North America and Eurasia, with coldspots in South America and Africa (Figure 1).

Coevolutionary signal in viral sharing

To test for the signal of evolutionary history in the viral sharing network, we analyzed two outcome variables (viral sharing as a binary state and as the total number of viruses shared) for two data structures (the entire pairwise host-host viral sharing matrix, or each hosts' sharing with *H. sapiens*; i.e., its role in zoonotic disease) in the pre- and post-imputation network. We analyzed these variables as a function of phylogenetic distance using generalized linear models (GLMs), with virus sharing coded as a binomial outcome (logit link) and the count data modeled using a Poisson distribution. GLMs were fit using the `stats` package in R, and adjusted R-squared values were derived using the `rsq` package. Model coefficients and significance are given in Tables 2 and 3. Response curves were finally plotted using the automated smoothing in the `ggplot` package with the same specifications.

We found that virus sharing, as a binary outcome, decoupled substantially from phylogeny after imputation. In large part, this can be explained by the fact that, with a 16-fold increase in connectance, binary sharing should become substantially less informative after imputation. (This also makes biological sense; for example, nearly all mammal species should share the capacity to be infected with true generalist viruses like rabies and influenza A.) In particular, the phylogenetic signal of virus sharing with humans became insignificant (p = 0.52) after imputation, the only insignificant relationship among those we tested. While the count data also recovered a reduction in effect size after imputation, we found that this reduction was much smaller and that the phylogenetic signal of sharing with *H. sapiens* was slightly more explanatory in the post-imputation network.

Community uniqueness analysis

We performed a measure of community compositional uniqueness using the local contribution to the beta-diversity approach[25] and specifically its extension to interaction data.[26] LCBD identifies locations (here, pixels) in which the community composition contributes more to the overall dissimilarity. For this section, we will note **X** the sites-by-items matrix, often referred to as a "community data matrix," in which locations are rows, and items (host, viruses, interactions) are columns. The total beta-diversity is measured as β = Var(**X**), after rows and columns with a marginal sum of 0 have been removed. The **X** matrix is then transformed by centering and squaring the values so that $\mathbf{S} = [S_{ij}] = [(X_{ij} - \overline{X_j})^2]$. The sum of squares in **X** is then simply given by $SS_{total} = \sum_i \sum_j S_{ij}$. From there, measuring the LCBD (i.e., the actual contribution of each location to β) is done by summing the matrix **X** row wise and dividing by the total sum of squares:

$$\mathrm{LCBD}_i = \sum_j \frac{S_{ij}}{SS_{total}}.$$

CellPress
OPEN ACCESS

Within every location, this value indicates the degree of uniqueness of this location (sampling unit) compared with all other sampling units in the data. LCBD values are typically, but not necessarily, measured after **X** has been transformed using Chord's or Hellinger's distance. This, however, assumes that sampling is close to complete, which is an unreasonable assumption in our observed dataset; because applying a Hellinger transformation post but not pre imputation would prevent a comparison of the results, we work on the raw matrices.

Prediction of zoonotic potential

We next tested whether the expanded host-range information available in the imputed network could improve zoonotic risk prediction in cases where information on individual viruses is limited. We expanded a recently developed model that combines summary statistics of viral genome composition and compositional similarity to human genes to predict zoonotic risk.[6]

We extracted pseudo-traits from the host-virus network using latent variables by extracting the left latent subspace of a random dot product graph decomposition.[59] We used the same number of dimensions (12) as for the low-rank approximation based on the imputation method; as LF-SVD functions as a dimensionality reduction technique (the ranks that are not considered for the imputation are essentially lost), the most conservative approach was to decide against re-adding dimensions for the zoonotic potential analysis. The feature matrix for viruses is given by

$$\overline{\mathbf{F}} = \mathbf{U}_{12}\sqrt{\mathbf{\Sigma}_{12}}$$

where $\mathbf{U}$ and $\mathbf{\Sigma}$ are the truncated left-subspace and singular values matrices of the decomposition of the network, respectively. This method was selected because the latent traits extracted this way can reproduce the original network within an arbitrary precision threshold and have been shown to capture the evolutionary signal on network structure.[59] To avoid leaking data on observed human infection into subsequent model training and evaluation steps, these network embeddings were generated while excluding humans. We also removed all viruses that had so far only been linked to humans because, after removal of humans from the network, these viruses were uniquely identifiable as some of the only included viruses with no links in the network (another potential data leak; a small number of viruses with no known mammalian hosts were similarly unlinked, but these were rare enough that a model that predicted all unlinked viruses as human-infecting would have had reasonably high performance).

Full genomes were available for 612 of the 681 remaining viruses. We used the reference sequence for each virus whenever available or the longest complete genome otherwise. These genomes were used to calculate the relevant genome composition measures described in Mollentze et al.[6] These were combined with the embeddings to train a series of gradient boosted classification and regression tree models to distinguish between viruses known to infect humans and other viruses. Viruses were randomly split into three datasets, using 70% for training, 15% for model calibration, and the remaining 15% for evaluating model performance.[6] This training/calibration/test procedure was repeated 1,000 times to assess variability in performance arising from current limited knowledge of the human-infecting virome. We compared models trained on either the original viral genome composition descriptors from Mollentze et al.[6] or a combination of viral genome composition and embeddings derived from either the observed network or from the imputed network. Finally, the best model by ROC-AUC (the model using viral genome features and embedding features describing the imputed network) was used to predict the probability of human infection for all 612 viruses. For this purpose, predictions were averaged across the best-performing 10% of models in which each virus occurred in the test data, a process akin to bagging.[30] Model performance was re-evaluated while excluding the virus being predicted to avoid selecting models based on their performance on the virus being predicted.

Feature importance was measured using their Shapley values, which measure the contribution of individual features to the final probability predicted for each virus.[60] We calculated the overall importance of each feature as the mean of absolute Shapley values across all viruses. When combined with features describing virus genomes, features derived from the t-SNE embedding of the SVD-imputed network tended to dominate (Figure 5). However, there was poor correspondence between embedding rank and relative feature importance (Spearman correlation = 0.315), highlighting the importance of including as much information about the network as possible.

SUPPLEMENTAL INFORMATION

Supplemental information can be found online at https://doi.org/10.1016/j.patter.2023.100738.

ACKNOWLEDGMENTS

This work was supported by funding to the Viral Emergence Research Initiative (VERENA) consortium, including NSF BII 2021909 and 2213854 and a grant from Institut de Valorisation des Données (IVADO). N.M. was funded by the Wellcome Trust (217221/Z/19/Z). T.P. and M.-A.O. were funded by the Fondation Courtois. This research was enabled in part by support provided by Calcul Québec (www.calculquebec.ca) and Compute Canada (www.computecanada.ca). We acknowledge that this study was conducted on land within the traditional unceded territory of the Saint Lawrence Iroquoian, Anishinabewaki, Mohawk, Huron-Wendat, and Omàmiwininiwak nations.

AUTHOR CONTRIBUTIONS

T.P. and C.J.C. conceived the study. T.P. and M.-A.O. performed the imputation. All authors contributed to data analysis and manuscript writing.

DECLARATION OF INTERESTS

The authors declare no competing interests.

Received: March 2, 2022
Revised: January 19, 2023
Accepted: March 31, 2023
Published: April 24, 2023

REFERENCES

1. Albery, G.F., Becker, D.J., Brierley, L., Brook, C.E., Christofferson, R.C., Cohen, L.E., Dallas, T.A., Eskew, E.A., Fagre, A., Farrell, M.J., et al. (2021). The science of the host–virus network. Nat. Microbiol. *6*, 1483–1492.
2. Dallas, T., Park, A.W., and Drake, J.M. (2017). Predicting cryptic links in host-parasite networks. PLoS Comput. Biol. *13*, e1005557.
3. Carlson, C.J., Zipfel, C.M., Garnier, R., and Bansal, S. (2019). Global estimates of mammalian viral diversity accounting for host sharing. Nat. Ecol. Evol. *3*, 1070–1075.
4. Carlson, C.J., Albery, G.F., Merow, C., Trisos, C.H., Zipfel, C.M., Eskew, E.A., Olival, K.J., Ross, N., and Bansal, S. (2022). Climate change increases cross-species viral transmission risk. Nature *607*, 555–562.
5. Nejati, M., Samavi, S., Derksen, H., and Najarian, K. (2016). Denoising by low-rank and sparse representations. J. Vis. Commun. Image Represent. *36*, 28–39. https://doi.org/10.1016/j.jvcir.2016.01.004.
6. Mollentze, N., Babayan, S.A., and Streicker, D.G. (2021). Identifying and prioritizing potential human-infecting viruses from their genome sequences. PLoS Biol. *19*, e3001390.
7. Stock, M., Poisot, T., Waegeman, W., and De Baets, B. (2017). Linear filtering reveals false negatives in species interaction data. Sci. Rep. *7*, 45908.
8. Gibb, R., Albery, G.F., Mollentze, N., Eskew, E.A., Brierley, L., Ryan, S.J., Seifert, S.N., and Carlson, C.J. (2022). Mammal virus diversity estimates are unstable due to accelerating discovery effort. Biol. Lett. *18*, 20210427.
9. Albery, G.F., Eskew, E.A., Ross, N., and Olival, K.J. (2020). Predicting the global mammalian viral sharing network using phylogeography. Nat. Commun. *11*, 2260.

10. Han, B.A., Schmidt, J.P., Bowden, S.E., and Drake, J.M. (2015). Rodent reservoirs of future zoonotic diseases. Proc. Natl. Acad. Sci. USA *112*, 7039–7044.
11. Carlson, C.J., Gibb, R.J., Albery, G.F., Brierley, L., Connor, R.P., Dallas, T.A., Eskew, E.A., Fagre, A.C., Farrell, M.J., Frank, H.K., et al. (2022). The global virome in one network (virion): an atlas of vertebrate-virus associations. mBio *13*, e0298521.
12. Farrell, M.J., Elmasri, M., Stephens, D.A., and Davies, T.J. (2022). Predicting missing links in global host–parasite networks. J. Anim. Ecol. *91*, 715–726.
13. Olival, K.J., Hosseini, P.R., Zambrana-Torrelio, C., Ross, N., Bogich, T.L., and Daszak, P. (2017). Host and viral traits predict zoonotic spillover from mammals. Nature *546*, 646–650.
14. Han, B.A., Kramer, A.M., and Drake, J.M. (2016). Global patterns of zoonotic disease in mammals. Trends Parasitol. *32*, 565–577.
15. Mollentze, N., and Streicker, D.G. (2020). Viral zoonotic risk is homogenous among taxonomic orders of mammalian and avian reservoir hosts. Proc. Natl. Acad. Sci. USA *117*, 9423–9430.
16. Luis, A.D., Hayman, D.T.S., O'Shea, T.J., Cryan, P.M., Gilbert, A.T., Pulliam, J.R.C., Mills, J.N., Timonin, M.E., Willis, C.K.R., Cunningham, A.A., et al. (2013). A comparison of bats and rodents as reservoirs of zoonotic viruses: are bats special? Proc. Biol. Sci. *280*, 20122753.
17. Wille, M., Geoghegan, J.L., and Holmes, E.C. (2021). How accurately can we assess zoonotic risk? PLoS Biol. *19*, e3001135.
18. Albery, G.F., Carlson, C.J., Cohen, L.E., Eskew, E.A., Gibb, R., Ryan, S.J., Sweeny, A.R., and Becker, D.J. (2022). Urban-adapted mammal species have more known pathogens. Nat. Ecol. Evol. *6*, 794–801.
19. Williams, R.J. (2011). Biology, methodology or chance? the degree distributions of bipartite ecological networks. PLoS One *6*, e17645.
20. Evans, M.V., Dallas, T.A., Han, B.A., Murdock, C.C., and Drake, J.M. (2017). Data-driven identification of potential zika virus vectors. Elife *6*, e22053.
21. Pandit, P.S., Anthony, S.J., Goldstein, T., Olival, K.J., Doyle, M.M., Gardner, N.R., Bird, B., Smith, W., Wolking, D., Gilardi, K., et al. (2023). Predicting the potential for zoonotic transmission and host associations for novel viruses. Commun. Biol. *6*, 25.
22. Stock, M., Piot, N., Vanbesien, S., Meys, J., Smagghe, G., and De Baets, B. (2021). Pairwise learning for predicting pollination interactions based on traits and phylogeny. Ecol. Model. *451*, 109508.
23. Wardeh, M., Blagrove, M.S.C., Sharkey, K.J., and Baylis, M. (2021). Divide-and-conquer: machine-learning integrates mammalian and viral traits with network features to predict virus-mammal associations. Nat. Commun. *12*, 3954.
24. Becker, D.J., Albery, G.F., Sjodin, A.R., Poisot, T., Bergner, L.M., Chen, B., Cohen, L.E., Dallas, T.A., Eskew, E.A., Fagre, A.C., et al. (2022). Optimising predictive models to prioritise viral discovery in zoonotic reservoirs. Lancet. Microbe *3*, e625–e637.
25. Legendre, P., and De Cáceres, M. (2013). Beta diversity as the variance of community data: dissimilarity coefficients and partitioning. Ecol. Lett. *16*, 951–963.
26. Poisot, T., Guéveneux-Julien, C., Fortin, M.-J., Gravel, D., and Legendre, P. (2017). Hosts, parasites and their interactions respond to different climatic variables. Glob. Ecol. Biogeogr. *26*, 942–951.
27. Forero, N., Muylaert, R.L., Seifert, S.N., Albery, G., Becker, D., Carlson, C.J., and Poisot, T. (2022). The coevolutionary mosaic of bat betacoronavirus emergence risk. Preprint at EcoEvoRxiv. https://doi.org/10.32942/osf.io/8mgv6.
28. Caraballo, D.A. (2022). Cross-species transmission of bat coronaviruses in the americas: contrasting patterns between alphacoronavirus and betacoronavirus. Microbiol. Spectr. *10*, e0141122.
29. Grange, Z.L., Goldstein, T., Johnson, C.K., Anthony, S., Gilardi, K., Daszak, P., Olival, K.J., O'Rourke, T., Murray, S., Olson, S.H., Togami, E., Vidal, G.; Expert Panel; PREDICT Consortium, and Mazet, J.A.K.; University of Edinburgh Epigroup members those who wish to remain anonymous (2021). Ranking the risk of animal-to-human spillover for newly discovered viruses. Proc. Natl. Acad. Sci. USA *118*. e2002324118. https://doi.org/10.1073/pnas.2002324118.
30. Babayan, S.A., Orton, R.J., and Streicker, D.G. (2018). Predicting reservoir hosts and arthropod vectors from evolutionary signatures in virus genomes. Science *362*, 577–580. https://doi.org/10.1126/science.aap9072.
31. Woolhouse, M.E.J., and Brierley, L. (2018). Epidemiological characteristics of human-infective RNA viruses. Sci. Data *5*, 180017. https://doi.org/10.1038/sdata.2018.17.
32. Ogunkoya, A.B., Beran, G.W., Umoh, J.U., Gomwalk, N.E., and Abdulkadir, I.A. (1990). Serological evidence of infection of dogs and man in Nigeria by lyssaviruses (family Rhabdoviridae). Trans. R. Soc. Trop. Med. Hyg. *84*, 842–845. https://doi.org/10.1016/0035-9203(90)90103-I.
33. Mendez-Rios, J.D., Martens, C.A., Bruno, D.P., Porcella, S.F., Zheng, Z.-M., and Moss, B. (2012). Genome sequence of erythromelalgia-related poxvirus identifies it as an ectromelia virus strain. PLoS One *7*, e34604.
34. Kuroya, M., and Ishida, N. (1953). Newborn virus pneumonitis (type Sendai). II. The isolation of a new virus possessing hemagglutinin activity. Yokohama Med. Bull. *4*, 217–233.
35. Maruyama, S. (1983). A survey on antibody against akabane virus in human beings and swine in Chiba Prefecture, Japan. J. Jpn. Vet. Med. Assoc. *36*, 330–333. https://doi.org/10.12935/jvma1951.36.330.
36. Centers for Disease Control CDC (1990). Update: filovirus infection in animal handlers. MMWR Morb. Mortal. Wkly. Rep. *39*, 221.
37. Olaleye, O.D., Omilabu, S.A., Ilomechina, E.N., and Fagbami, A.H. (1990). A survey for haemagglutination-inhibiting antibody to west nile virus in human and animal sera in Nigeria. Comp. Immunol. Microbiol. Infect. Dis. *13*, 35–39.
38. L'vov, D.K., Kostiukova, M.A., Pak, T.P., and Gromashevskiĭ, V.L. (1980). Isolation of an arbovirus antigenically related to Issyk-Kul virus from the blood of a human patient. Vopr. Virusol. 61–62.
39. Poisot, T., Carlson, C.J., Mollentze, N., and Farrell, M.J. (2021). viralemergence/trefle: v0.0.1. https://doi.org/10.5281/zenodo.4850581.
40. Mollentze, N. (2021). viralemergence/haystack: v0.0.1. https://doi.org/10.5281/zenodo.4850643.
41. Gibb, R., Albery, G.F., Becker, D.J., Brierley, L., Connor, R., Dallas, T.A., Eskew, E.A., Farrell, M.J., Rasmussen, A.L., Ryan, S.J., et al. (2021). Data proliferation, reconciliation, and synthesis in viral ecology. Preprint at bioRxiv. https://doi.org/10.1101/2021.01.14.426572.
42. Wardeh, M., Risley, C., McIntyre, M.K., Setzkorn, C., and Baylis, M. (2015). Database of host-pathogen and related species interactions, and their global distribution. Sci. Data *2*, 150049.
43. Stephens, P.R., Pappalardo, P., Huang, S., Byers, J.E., Farrell, M.J., Gehman, A., Ghai, R.R., Haas, S.E., Han, B., Park, A.W., et al. (2017). Global mammal parasite database version 2.0. Ecology *98*, 1476.
44. Shaw, L.P., Wang, A.D., Dylus, D., Meier, M., Pogacnik, G., Dessimoz, C., and Balloux, F. (2020). The phylogenetic range of bacterial and viral pathogens of vertebrates. Mol. Ecol. *29*, 3361–3379.
45. Strydom, T., Dalla Riva, G.V., and Poisot, T. (2020). Svd entropy reveals the high complexity of ecological networks. Preprint at EcoEvoRxiv. https://doi.org/10.32942/osf.io/q9v85.
46. Shlens, J. (2014). A tutorial on principal component analysis. Preprint at arXiv. https://doi.org/10.48550/arXiv.1404.1100.
47. Bezanson, J., Karpinski, S., Shah, V.B., and Edelman, A. (2012). Julia: a fast dynamic language for technical computing. Preprint at arXiv. https://doi.org/10.48550/arXiv.1209.5145.
48. Poisot, T., Bélisle, Z., Hoebeke, L., Stock, M., and Szefer, P. (2019). Ecologicalnetworks. jl: analysing ecological networks of species interactions. Ecography *42*, 1850–1861.
49. Upham, N.S., Esselstyn, J.A., and Jetz, W. (2019). Inferring the mammal tree: species-level sets of phylogenies for questions in ecology, evolution, and conservation. PLoS Biol. *17*, e3000494.

CellPress
OPEN ACCESS

50. Winter, D. (2017). rentrez: An r package for the ncbi eutils api. R J. *9*, 520–526.
51. Teitelbaum, C.S., Amoroso, C.R., Huang, S., Davies, T.J., Rushmore, J., Drake, J.M., Stephens, P.R., Byers, J.E., Majewska, A.A., and Nunn, C.L. (2020). A comparison of diversity estimators applied to a database of host–parasite associations. Ecography *43*, 1316–1328.
52. Nunn, C.L., Altizer, S., Jones, K.E., and Sechrest, W. (2003). Comparative tests of parasite species richness in primates. Am. Nat. *162*, 597–614.
53. Ezenwa, V.O., Price, S.A., Altizer, S., Vitone, N.D., and Cook, K.C. (2006). Host traits and parasite species richness in even and odd-toed hoofed mammals, artiodactyla and perissodactyla. Oikos *115*, 526–536.
54. Huang, S., Drake, J.M., Gittleman, J.L., and Altizer, S. (2015). Parasite diversity declines with host evolutionary distinctiveness: a global analysis of carnivores. Evolution *69*, 621–630.
55. Freckleton, R., Harvey, P., and Pagel, M. (2002). Phylogenetic analysis and comparative data: a test and review of evidence. Am. Nat. *160*, 712–726.
56. Orme, D., Freckleton, R., Thomas, G., Petzoldt, T., Fritz, S., Isaac, N., and Pearse, W. (2012). Caper: comparative analyses of phylogenetics and evolution in r. R package version 0.5 *2*, 458.
57. Pagel, M. (1999). Inferring the historical patterns of biological evolution. Nature *401*, 877–884.
58. Washburne, A.D., Silverman, J.D., Morton, J.T., Becker, D.J., Crowley, D., Mukherjee, S., David, L.A., and Plowright, R.K. (2019). Phylofactorization: a graph partitioning algorithm to identify phylogenetic scales of ecological data. Ecol. Monogr. *89*, e01353.
59. Dalla Riva, G.V., and Stouffer, D.B. (2016). Exploring the evolutionary signature of food webs' backbones using functional traits. Oikos *125*, 446–456.
60. Lundberg, S.M., and Lee, S.-I. (2017). A unified approach to interpreting model predictions. Adv. Neural Inf. Process. Syst. *30*, 4765–4774.

Patterns, Volume *4*

Supplemental information

Network embedding unveils

the hidden interactions in the mammalian virome

Timothée Poisot, Marie-Andrée Ouellet, Nardus Mollentze, Maxwell J. Farrell, Daniel J. Becker, Liam Brierley, Gregory F. Albery, Rory J. Gibb, Stephanie N. Seifert, and Colin J. Carlson

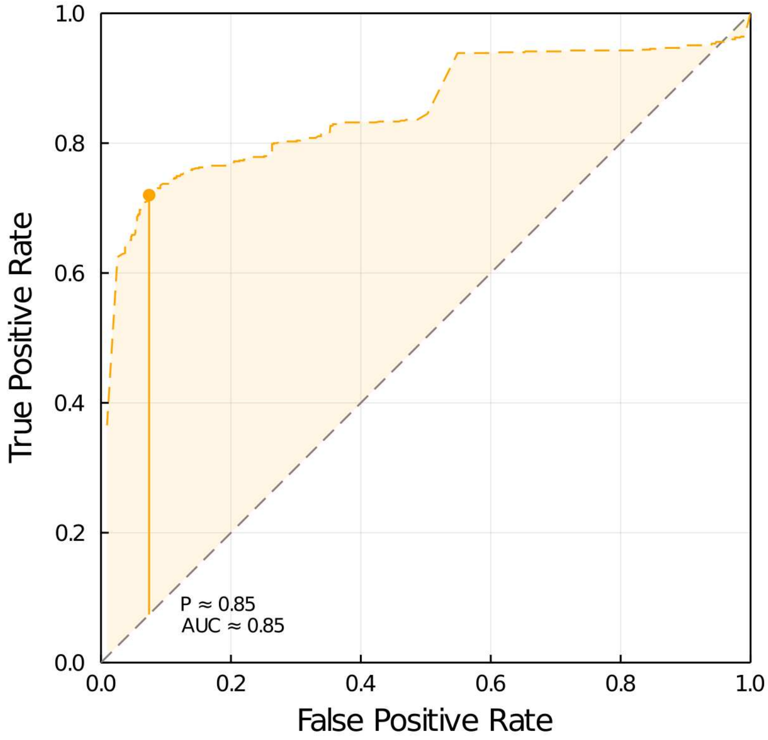

Figure S1: **Receiver operating characteristic (ROC) curve for the best model**. All models (from SVD rank 1 to 20, and using three linear filtering parameterization) have been compared on the same training/validation dataset by measuring the area under the ROC curve. The best model has an area under the ROC curve (AUC) of 0.85. The threshold turning the continuous prediction of LF-SVD into a binary classification was picked to simultaneously maximize the true positive rate and minimize the false positive rate, in practice by maximizing Youden's informedness. By coincidence, this threshold is also approximately equal to 0.85 in the best model.

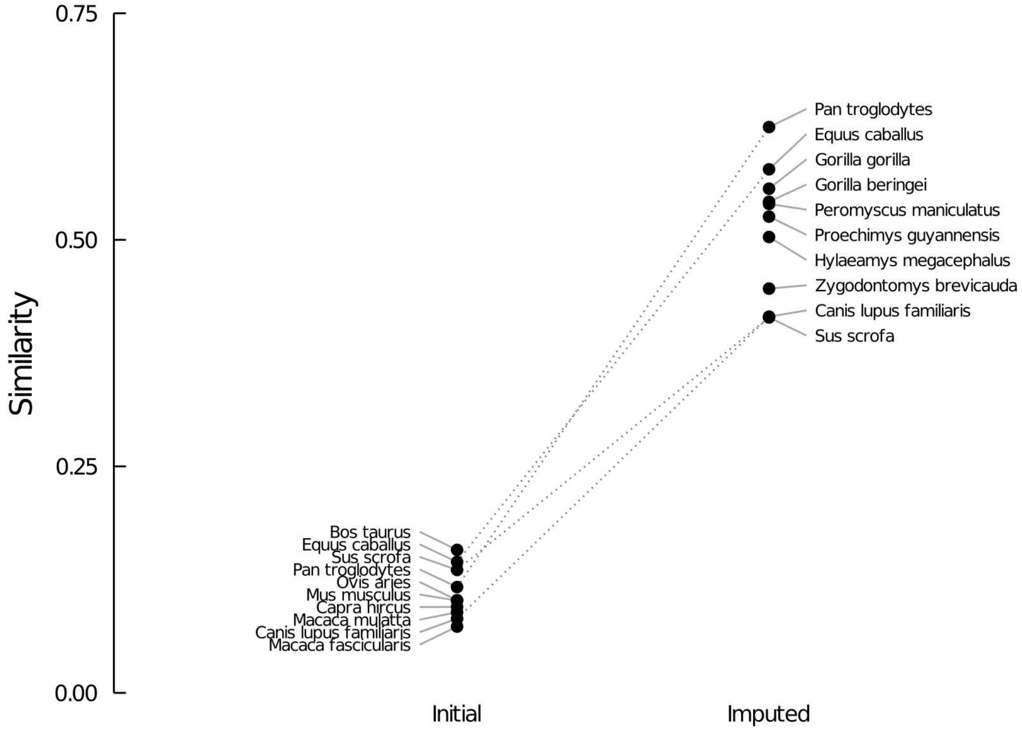

Figure S2: **Pairwise host-similarity to *Homo sapiens* changes post imputation**. The ten hosts with the most viral overlap to *Homo sapiens* (as measured by Jaccard similarity) tend to be livestock. By contrast, the ten most similar hosts after imputation are mostly composed of primates and rodents, which suggests that LF-SVD is able to overcome taxonomic bias in the original dataset.

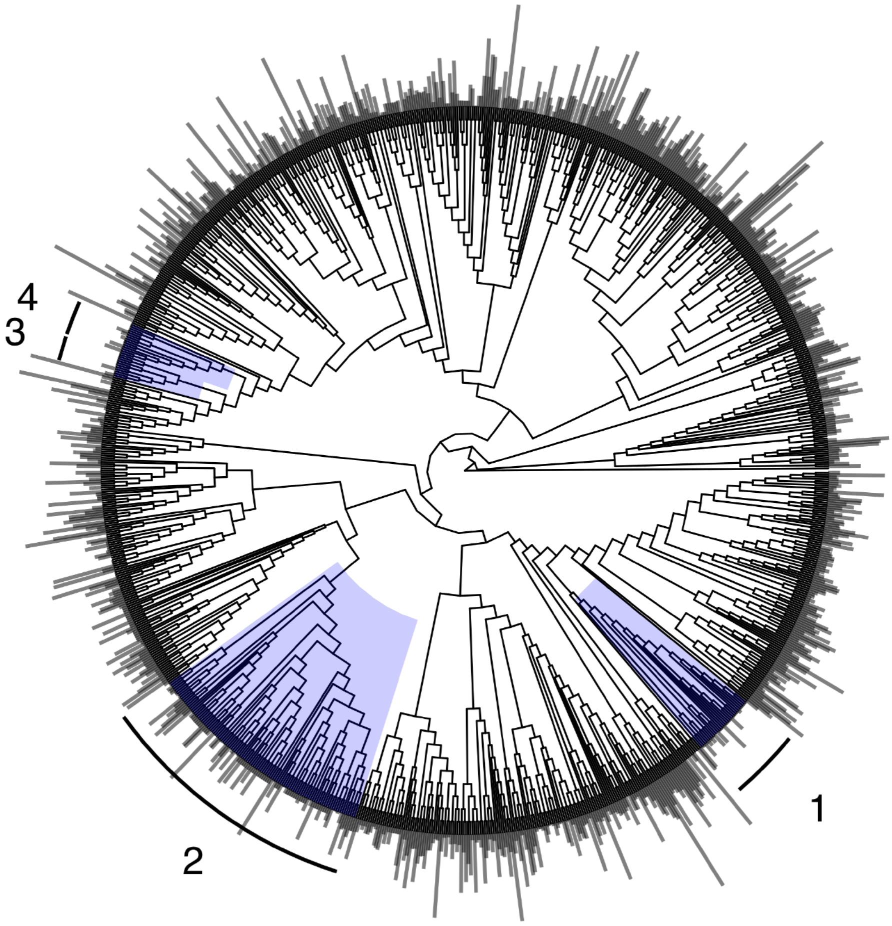

Figure S3: **Phylogenetic bias in missing viruses.** Phylogenetic factorization determined that the majority of species have no phylogenetic signal in the number of missing viruses estimated by the LF-SVD model, with the exception of a handful of small clades that included cetaceans (clade 1), a mostly insectivorous subclade of the Yangochiroptera (clade 2), and two small rodent clades (clades 3 and 4), all of which have significantly fewer than average.

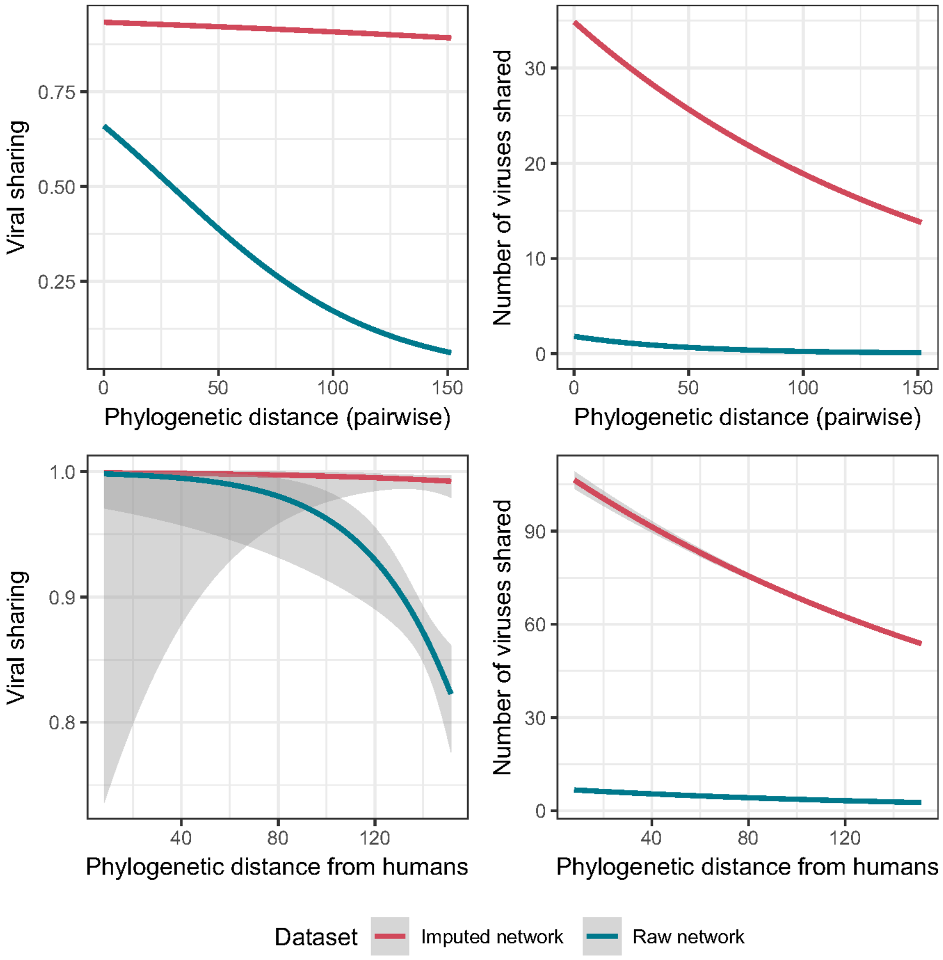

Figure S4: **Evolutionary signals dominate viral sharing.** Phylogenetic distance among all mammals (top) or from humans (bottom) structure viral sharing measured as a binary trait (left) or based on the total number of shared viruses (right). In the imputed network, mosts hosts have many more viruses; as a result, phylogenetic distance is less informative of whether hosts share viruses, because most hosts share at least one virus, but the phylogenetic signal of the count data is much stronger. Curves are given as generalized linear model smooths, with a Poisson distribution for count data and a binomial distribution with a logit link function for viral sharing.

Table S1: **Model performance for the top 10 models by AUC.** Metrics include the AUC and cutoff (expressed as a pseudo-probability), the true positive and true negative rates (TPR, TNR), the positive and negative predictive values (PPV, NPV), the false negative and positive rates (FNR, FPR), the false discovery and false omission rates (FDR, FOR), the critical success index (CSI), accuracy (ACC), and Youden's J.

model	rank	AUC	cutoff	TPR	TNR	PPV	NPV
connectance	12	0.849	0.846	0.72	0.925	0.906	0.769
connectance	11	0.846	0.908	0.684	0.936	0.914	0.75
connectance	17	0.844	0.929	0.692	0.935	0.913	0.754
connectance	8	0.842	0.705	0.701	0.895	0.868	0.751
hybrid	12	0.841	0.707	0.703	0.877	0.851	0.748
connectance	14	0.839	0.902	0.7	0.929	0.907	0.758
hybrid	11	0.837	0.82	0.647	0.918	0.888	0.723
connectance	5	0.836	0.931	0.66	0.94	0.916	0.735
connectance	7	0.836	0.948	0.655	0.957	0.939	0.735
connectance	16	0.835	0.961	0.667	0.945	0.923	0.741

(Continued:)

model	rank	FNR	FPR	FDR	FOR	CSI	ACC	J
connectance	12	0.28	0.074	0.093	0.23	0.669	0.823	0.645
connectance	11	0.315	0.063	0.085	0.25	0.643	0.811	0.621
connectance	17	0.307	0.064	0.086	0.245	0.649	0.814	0.627
connectance	8	0.298	0.104	0.131	0.248	0.634	0.798	0.596
hybrid	12	0.296	0.122	0.148	0.251	0.626	0.79	0.581
connectance	14	0.299	0.07	0.092	0.241	0.653	0.815	0.629
hybrid	11	0.352	0.081	0.111	0.276	0.598	0.783	0.566
connectance	5	0.339	0.059	0.083	0.264	0.623	0.8	0.6
connectance	7	0.344	0.042	0.06	0.264	0.628	0.806	0.613
connectance	16	0.332	0.054	0.076	0.258	0.632	0.807	0.613

Table S2: **Imputation reduces the effect of sampling bias.** To explore whether network imputation via LF-SVD is extrapolating existing research biases, we conducted a set of comparative analyses investigating the how the explanatory power of sampling effort on viral species richness changes after network imputation. We find that after imputation, the slope of the relationship (β) decreases, and sampling effort explains less of the variance in viral richness (R^2), suggesting that imputation via LF-SVD is not merely recapitulating the observed sampling effort per host. Statistics are given for a phylognetic generalized linear model fit with the maximum likelihood estimate of Pagel's λ. Predictors and responses were log-10 transformed prior to analyses.

Viral richness	Publications	β	S.E.	R^2	λ	λ 95% CI
Observed	Cites (all)	0.53	0.02	0.46	0.59	(0.47-0.69)
LF-SVD imputed	Cites (all)	0.39	0.02	0.23	0.59	(0.45-0.72)
Observed	Cites (virus-related)	0.71	0.02	0.54	0.45	(0.31-0.58)
LF-SVD imputed	Cites (virus-related)	0.47	0.03	0.22	0.60	(0.46-0.71)

Table S3: **Phylofactorization of missing viruses.** Significant clades identified from a phylogenetic factorization of missing virus counts. Included taxa are listed alongside the number of species and the mean number of missing viruses for each clade in comparison to the paraphyletic remainder. Clade codes match ED Figure S3.

c

Clade	Included taxa	n	clade
1	Ziphiidae, Physeteridae, Phocoenidae, Monodontidae, Delphinidae, Eschrichtiidae, Balaenopteridae, Balaenidae	30	18
2	Nycteridae, Emballonuridae, Natalidae, Molossidae, Vespertilionidae	109	43
3	*Calomys*, *Graomys*, *Phyllotis*, *Loxodontomys*, *Abrothrix*	11	11
4	*Bibimys*, *Oxymycterus*, *Necromys*, *Akodon*, *Thaptomys*	15	16
